# Epidemiology and Costs of Postsepsis Morbidity, Nursing Care Dependency, and Mortality in Germany, 2013 to 2017

**DOI:** 10.1001/jamanetworkopen.2021.34290

**Published:** 2021-11-12

**Authors:** Carolin Fleischmann-Struzek, Norman Rose, Antje Freytag, Melissa Spoden, Hallie C. Prescott, Anna Schettler, Lisa Wedekind, Bianka Ditscheid, Josephine Storch, Sebastian Born, Peter Schlattmann, Christian Günster, Konrad Reinhart, Christiane S. Hartog

**Affiliations:** 1 Center for Sepsis Control and Care, Jena University Hospital, Friedrich Schiller University Jena, Jena, Germany; 2 Institute of Infectious Diseases and Infection Control, Jena University Hospital, Jena, Germany; 3 Institute of General Practice and Family Medicine, Jena University Hospital, Jena, Germany; 4 Research Institute of the Local Health Care Funds, Berlin, Germany; 5 Department of Medicine, University of Michigan, Ann Arbor; 6 Department for Anesthesiology and Intensive Care Medicine, Jena University Hospital, Jena, Germany; 7 Institute of Medical Statistics, Computer and Data Sciences, Jena University Hospital, Jena, Germany; 8 Department of Anesthesiology and Operative Intensive Care Medicine, Charité Universitätsmedizin Berlin, Berlin, Germany; 9 Klinik Bavaria, Kreischa, Germany; 10 VA Center for Clinical Management Research, Ann Arbor, Michigan

## Abstract

**Importance:**

Sepsis survivorship is associated with postsepsis morbidity, but epidemiological data from population-based cohorts are lacking.

**Objective:**

To quantify the frequency and co-occurrence of new diagnoses consistent with postsepsis morbidity and mortality as well as new nursing care dependency and total health care costs after sepsis.

**Design, Setting, and Participants:**

This retrospective cohort study based on nationwide health claims data included a population-based cohort of 23.0 million beneficiaries of a large German health insurance provider. Patients aged 15 years and older with incident hospital-treated sepsis in 2013 to 2014 were included. Data were analyzed from January 2009 to December 2017.

**Exposures:**

Sepsis, identified by *International Statistical Classification of Diseases and Related Health Problems, Tenth Revision* (*ICD-10*) hospital discharge codes.

**Main Outcomes and Measures:**

New medical, psychological, and cognitive diagnoses; long-term mortality; dependency on nursing care; and overall health care costs in survivors at 1 to 12, 13 to 24, and 25 to 36 months after hospital discharge.

**Results:**

Among 23.0 million eligible individuals, we identified 159 684 patients hospitalized with sepsis in 2013 to 2014. The mean (SD) age was 73.8 (12.8) years, and 75 809 (47.5%; 95% CI, 47.2%-47.7%) were female patients. In-hospital mortality was 27.0% (43 177 patients; 95% CI, 26.8%-27.3%). Among 116 507 hospital survivors, 86 578 (74.3%; 95% CI, 74.1%-74.6%) had a new diagnosis in the first year post sepsis; 28 405 (24.4%; 95% CI, 24.1%-24.6%) had diagnoses co-occurring in medical, psychological, or cognitive domains; and 23 572 of 74 878 survivors (31.5%; 95% CI, 31.1%-31.8%) without prior nursing care dependency were newly dependent on nursing care. In total, 35 765 survivors (30.7%; 95% CI, 30.4%-31.0%) died within the first year. In the second and third year, 53 089 (65.8%; 95% CI, 65.4%-66.1%) and 40 959 (59.4%; 95% CI, 59.0%-59.8%) had new diagnoses, respectively. Health care costs for sepsis hospital survivors for 3 years post sepsis totaled a mean of €29 088/patient ($32 868/patient) (SD, €44 195 [$49 938]). New postsepsis morbidity (>1 new diagnosis) was more common in survivors of severe sepsis (75.6% [95% CI, 75.1%-76.0%]) than nonsevere sepsis (73.7% [95% CI, 73.4%-74.0%]; *P* < .001) and more common in survivors treated in the intensive care unit (78.3% [95% CI, 77.8%-78.7%]) than in those not treated in the intensive care unit (72.8% [95% CI, 72.5%-73.1%]; *P* < .001). Postsepsis morbidity was 68.5% (95% CI, 67.5%-69.5%) among survivors without prior morbidity and 56.1% (95% CI, 54.2%-57.9%) in survivors younger than 40 years.

**Conclusions and Relevance:**

In this study, new medical, psychological, and cognitive diagnoses consistent with postsepsis morbidity were common after sepsis, including among patients with less severe sepsis, no prior diagnoses, and younger age. This calls for more efforts to elucidate the underlying mechanisms, define optimal screening for common new diagnoses, and test interventions to prevent and treat postsepsis morbidity.

## Key Points

**Question:**

How common are new and co-occurring medical, cognitive, or psychological diagnoses, new nursing care dependency, and postacute mortality among individuals who survive sepsis?

**Findings:**

In this cohort study of 116 507 survivors of hospital-treated sepsis in Germany, nearly three-quarters had new medical, cognitive, or psychological diagnoses; nearly one-third were newly dependent on nursing care; and more than of 3 in 10 died in first year post sepsis. New diagnoses co-occurred in one-quarter of participants and affected sepsis survivors irrespective of preexisting diagnoses, sepsis severity, and intensive care unit treatment.

**Meaning:**

These findings suggest that postsepsis morbidity may be more common and severe than previously believed, calling for increased efforts to prevent and treat the sequelae of severe infections.

## Teaser

This cohort study evaluates the frequency and co-occurrence of new diagnoses consistent with postsepsis morbidity and mortality as well as new nursing care dependency and total health care costs after sepsis.

## Introduction

Sepsis is a disability-inducing event, resulting in considerable financial burden for health care systems.^1,2,3^ An estimated 38 million patients survive sepsis each year,^4^ many of whom experience persisting health problems,^1,5^ including new or worsened physical,^6^ psychological,^7,8^ and cognitive^6^ impairments. Because of these sequelae, sepsis survivors often need ongoing nursing care and experience increased risk of death.^9^

While the long-term consequences of sepsis are increasingly recognized, there are limited epidemiologic data on the co-occurrence of sepsis sequelae and the rate of sequelae in younger patients or those with less severe sepsis. In a nationwide US cohort of older sepsis survivors, one-sixth experienced persistent physical disability or cognitive impairment, and one-third died during the following year.^6,10^ In survivors treated in the intensive care unit (ICU), there seems to be a considerable overlap with post–intensive care syndrome.^11^ However, 50% of severe sepsis patients in the United States^12^ and two-thirds of patients hospitalized with sepsis in Germany^13^ are not treated in an ICU. Data on sepsis sequelae in this patient group are lacking. This study aimed to (1) quantify the frequency and co-occurrence of new medical, psychological, and cognitive diagnoses consistent with postsepsis morbidity; (2) compare the rates of mortality and new diagnoses by age group, sepsis severity, ICU treatment, and preexisting diseases; and (3) measure the cumulative costs of care.

## Methods

The study was preregistered (DRKS00016340) and approved by the Jena University Hospital institutional review board. The requirement for informed consent was waived because all data were deidentified. This study was reported according to the Strengthening the Reporting of Observational Studies in Epidemiology (STROBE) reporting guideline.

### Data Source

We performed a longitudinal population-based cohort study using deidentified health claims data from the health insurer Allgemeine Ortskrankenkasse (AOK) from 2009 to 2017. Health insurance is mandatory in Germany; residents select any insurer and enroll without restriction. AOK is the largest nationwide health insurer, covering approximately 30% of the German population.^14^

### Identification of Patients With Sepsis

Patients aged 15 years and older with an inpatient hospitalization for sepsis (discharged January 1, 2013, to December 31, 2014) were identified by explicit *International Statistical Classification of Diseases and Related Health Problems, Tenth Revision, German Modification *(*ICD-10-GM*) codes for sepsis coded as primary or secondary discharge diagnoses (eAppendix in the Supplement). We defined and stratified sepsis severity according to the sepsis 1 and sepsis 2 definitions^15,16^ as sepsis, indicating all forms; severe sepsis or septic shock; and nonsevere sepsis. Coding of sepsis in Germany is rigorously controlled by the Medical Service of the Health Funds; coding of nonsevere sepsis was restricted to cases with positive blood culture and at least 2 Systemic Inflammatory Response Syndrome (SIRS) criteria or to cases with 4 SIRS criteria in case of negative blood cultures, according to German coding regulations during the complete observation period.^13,17^ The first hospitalization with sepsis was defined as the index hospitalization. We excluded patients with a diagnosis of sepsis in the 2 years preceding hospitalization. Preexisting diagnoses and comorbidities were assessed in a period for as long as 12 months (or 5 years for asplenia) prior to hospitalization. Therefore, patients who were not continuously insured from January 1, 2009, through the 3-year follow-up period after the index hospitalization (or until death) were excluded.

### Characteristics of Patients With Sepsis

Patient demographics and clinical characteristics were assessed based on hospital discharge data as well as a 12-month look-back in inpatient and outpatient claims. Study definitions are presented in the eAppendix in the Supplement. Prior nursing home residency and dependency on nursing care were determined based on graded care needs (entitling patients to long-term care insurance benefits, which include care provided by informal or formal caregivers or nursing home placement).^18^

### Determining New Diagnoses and Costs

Based on a comprehensive literature review on postsepsis morbidity, we identified relevant diagnoses consistent with postsepsis morbidity. To translate diagnoses to *ICD-10-GM* codes, we adapted established definitions^19,20,21^ and complemented them by own searches (eAppendix in the Supplement). Experts from intensive care, internal medicine, neurology, psychiatry, family medicine, and rehabilitation medicine reviewed and approved the list of diagnoses consistent with postsepsis morbidity. Diagnoses were grouped into 3 domains (ie, medical, psychological, and cognitive) denoting 3 categories of clinical sequelae.^9^ The medical domain included respiratory, cardiovascular, cerebrovascular, kidney, hepatic, metabolic, urogenital, and neuromuscular/musculoskeletal diagnoses, sensory disorders, anemia, fatigue, decubitus ulcer, pain, multidrug-resistant infections, complications of the tracheostomy, and impairments of nutrition. The psychological domain included depression, anxiety, posttraumatic stress disorder (PTSD), sleeping disorders, and substance use disorders. The cognitive domain included mild to severe cognitive impairment as a single diagnosis. We also assessed nursing care dependency and postdischarge mortality. Finally, we used *ICD-10-GM* and *Operationen- und Prozedurenschlüssel *(*OPS*) codes to assess long-term mechanical ventilation and dialysis (eAppendix in the Supplement).

We determined the prevalence of each diagnosis after sepsis during the 1 to 12, 13 to 24, and 25 to 36 months after index hospital discharge among hospital survivors, 12-month survivors, and 24-month survivors, respectively. Diagnoses were considered present if at least 1 of the *ICD-10-GM* codes for the diagnosis was reported during a hospitalization or outpatient visit after the index hospital discharge. Diagnoses were considered new if there was no related *ICD-10-GM* code during the preceding observation period (12-month look-back: first year diagnoses; first year: second year diagnoses; second year: third year diagnoses) in inpatient and outpatient claims data. Survivors who did not have the particular diagnosis during the preceding time period were considered at-risk for incident diagnoses. Thus, incidence of each diagnosis in the first year post sepsis was measured only in patients at risk (ie, did not have the diagnosis in the year before sepsis diagnosis). Likewise, incident diagnoses in the second- and third-year survivors were measured among patients without the diagnosis through the first and second years, respectively. Total health care costs were measured from a health insurance perspective and calculated per patient as sum of costs of hospitalizations, outpatient consultations, medication prescriptions, treatment prescriptions (eg, physical therapy) and rehabilitation.

### Statistical Analysis

We reported continuous variables with means and SDs and medians and IQRs and categorical variables by proportions and 95% CIs. We used χ^2^ test and Welch-Satterthwaite *t* test for comparisons between subgroups. All reported *P* values refer to 2-sided tests with a statistical significance level of α = .05. For all descriptive and inferential statistical analyses, SAS Enterprise Guide version 7.1 (SAS Institute) was used. Kaplan-Meier estimates of the survivor functions were used for survival analyses. Differences in the survivor functions between subpopulations were tested with the log-rank test. To facilitate the interpretation of survivor functions, nonparametric estimates of hazard functions based on B-splines with 95% confidence bands are provided.^22^ The software R version 4.1.0 was used for all survival analyses by means of the R packages survival^23,24^ and bshazard^25^ (R Project for Statistical Computing).

## Results

### Index Population

**Figure 1.**
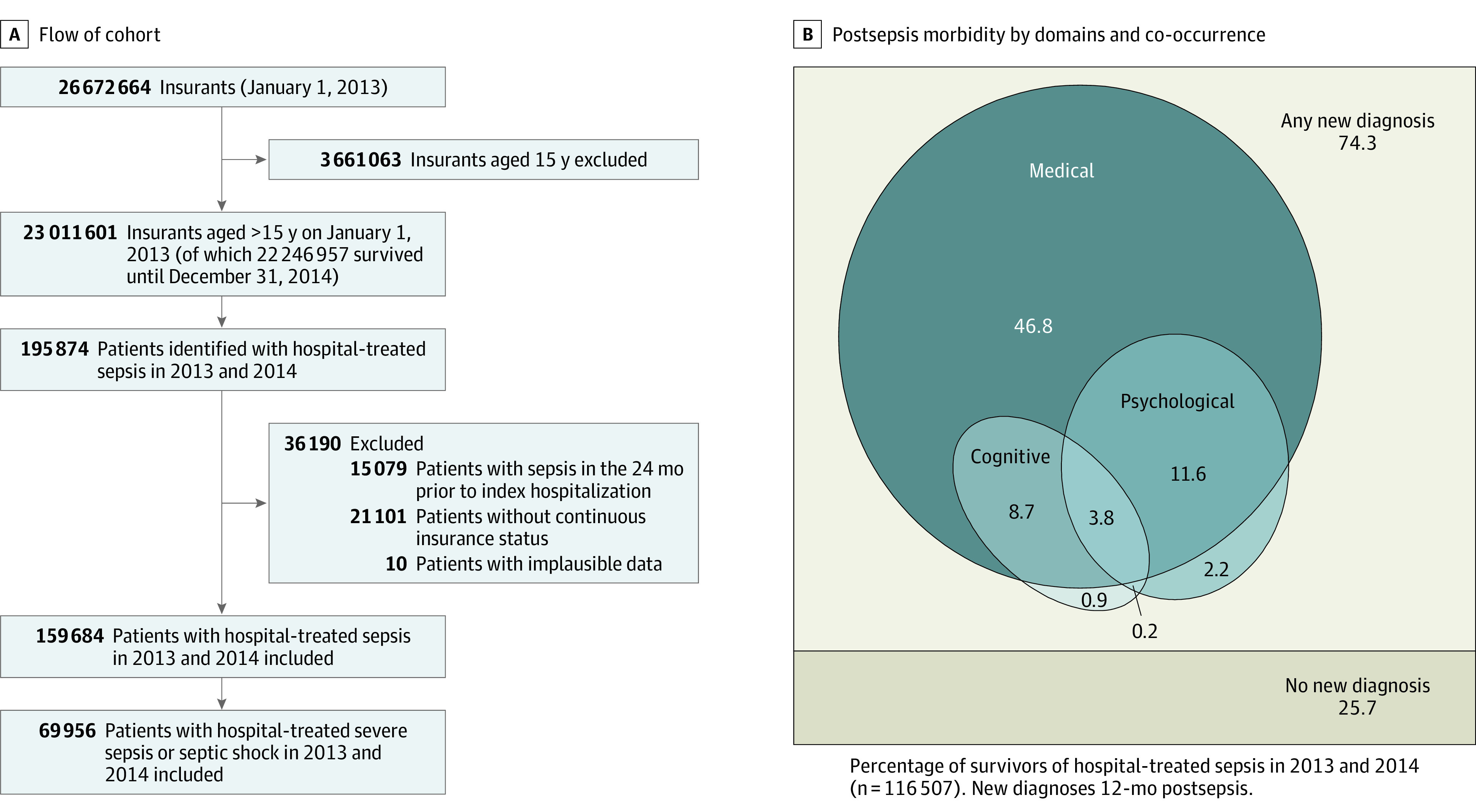
Study Flowchart and Postsepsis Morbidity by Domains and Co-occurrence B, This Euler diagram shows the proportion of survivors with new medical, cognitive, or psychological diagnoses in the first year.

Among 23.0 million eligible individuals, there were 159 684 index sepsis hospitalizations from 2013 to 2014 (353 per 100 000 person-years) (Figure 1A). Patients with sepsis had a mean (SD) age of 73.8 (12.8) years, 75 809 (47.5%; 95% CI, 47.2%-47.7%) were female patients, and had modest comorbidity burden (mean [SD] unweighted Charlson Comorbidity Index, 2.1 [1.5]). In the 12 months prior to sepsis, 61 167 (38.3%; 95% CI, 38.1%-38.5%) were dependent on nursing care, and 18 636 (11.7%; 95% CI, 11.5%-11.8%) resided in nursing homes. Only 10 666 (6.7%; 95% CI, 6.6%-6.8%) had no preexisting medical, psychological, or cognitive diagnoses in the 12 months before sepsis. Overall, 20 144 (12.6%; 95% CI, 12.5%-12.8%) were employed prior to hospitalization.

Of 159 684 sepsis hospitalizations, 54 317 (34.0%) received intensive care; 69 956 (43.8%) had severe sepsis, including 20 589 (29.4%) with septic shock. Patients with sepsis were hospitalized for a mean (SD) 20.6 (20.8) days, and in-hospital mortality was 27.0% (43 177 patients; 95% CI, 26.8%-27.3%) (eTable 1 in the Supplement). In-hospital mortality was higher in patients treated in the ICU vs those who were not (22 079 [40.6%; 95% CI, 40.2%-41.1%] vs 21 098 [20.0%; 95% CI, 19.8%-20.3%]; *P* < .001), was higher for those with severe vs nonsevere sepsis (32 116 [45.9%; 95% CI, 45.5%-46.3%] vs 11 061 [12.3%; 12.1%-12.5%]; *P* < .001), and highest in those with septic shock (12 701 [61.7%; 95% CI, 61.0%-62.4%) (eTable 2 in the Supplement). Patients with no preexisting medical, psychological, or cognitive diagnoses had an in-hospital mortality of 19.2% (2044 patients; 95% CI, 18.4%-19.9%). Overall, 6397 hospital survivors (5.5%; 95% CI, 5.4%-5.6%) were discharged to postacute inpatient rehabilitation. Demographic and clinical features of subgroups are reported in eTable 2 in the Supplement.

### New Diagnoses and Care Dependency in Sepsis Survivors

Of the 116 507 patients who survived index hospitalization, 86 578 (74.3%; 95% CI, 74.1%-74.6%) had a new medical, psychological, or cognitive diagnosis consistent with postsepsis morbidity during their first year post discharge. Specifically, 82 629 (70.9%; 95% CI, 70.7%-71.2%) had a new medical diagnosis, 20 840 (17.9%; 95% CI, 17.7%-18.1%) had a new psychological diagnosis, and 15 955 of 86 350 at-risk survivors (18.5%; 95% CI, 18.2%-18.7%) had a new cognitive diagnosis (Table 1).^26^ Among 74 878 hospital survivors without prior nursing care dependency, 23 572 (31.5%; 95% CI, 31.1%-31.8%) had new nursing care dependency during the first year post sepsis, 1890 of 115 025 at-risk survivors (1.6%; 95% CI, 1.6%-1.7%) required new long-term mechanical ventilation, and 3144 of 111 993 at-risk survivors (2.8%; 95% CI, 2.7%-2.9%) required new dialysis.

**Table 1.** Postsepsis Diagnoses, Mortality, and Costs Over 3 Years^a^

Outcomes among all survivors at start of the time period	Survivors, by follow-up from index hospital discharge					
	1-12 mos		13-24 mos		25-36 mos	
	No. (n = 116 507)	% (95% CI)	No. (n = 80 742)	% (95% CI)	No. (n = 68 940)	% (95% CI)
Any new diagnosis^b^	86 578	74.3 (74.1-74.6)	53 089	65.8 (65.4-66.1)	40 959	59.4 (59.0-59.8)
New medical diagnosis	82 629	70.9 (70.7-71.2)	49 486	61.3 (61.0-61.6)	37885	55.0 (54.6-55.3)
New medical diagnoses, No.						
Mean (SD)	1.9 (1.9)	NA	1.4 (1.6)	NA	1.4 (1.6)	NA
Median (IQR)	1 (0-3)	NA	1 (0-2)	NA	1 (0-2)	NA
New psychological diagnosis	20 840	17.9 (17.7-18.1)	10 296	12.8 (12.5 – 13.0)	8429	12.2 (12.0-12.5)
New psychological diagnoses, No.						
Mean (SD)	1.2 (0.5)	NA	1.2 (0.4);	NA	1.1 (0.4)	NA
Median (IQR)	1 (1-1)	NA	1 (1-1)	NA	1 (1-1)	NA
New cognitive diseases, No./No. at risk	15 955/86 350	18.5 (18.2-18.7)	5383/55 144	9.8 (9.5 – 10.)	4807/48 909	9.8 (9.6-10.1)
New mechanical ventilation, No./No. at risk	1890/115 025	1.6 (1.6-1.7)	906/78 999	1.1 (1.1-1.2)	751/67 531	1.1 (1.0-1.2)
New dialysis, No./No. at risk	3144/111 993	2.8 (2.7-2.9)	1040/76 863	1.4 (1.3-1.4)	789/65 925	1.2 (1.1-1.3)
New nursing home residence, No./No. at risk	12 485/103 912	12.0 (11.8-12.2)	2223/66 502	3.3 (3.2-3.5)	1950/57 409	3.4 (3.3-3.5)
New dependency on nursing care, No./No. at risk^c^	23 572/74 878	31.5 (31.1-31.8)	3784/40 925	9.2 (9.0-9.5)	4272/36 166	11.8 (11.5-12.1)
Mortality	35 765	30.7 (30.4-31.0)	11 802	14.6 (14.4-14.9)	9082	13.2 (12.9-13.4)
Total health care costs, €^d^						
Mean (SD)	14 891 (24 737)	NA	11 503 (20 788)	NA	10 521 (19 146)	NA
Median (IQR)	7055 (2422-17 379)	NA	5040 (1909-12 813)	NA	4607 (1771-11 573)	NA

Abbreviation: NA, not applicable.

^a^ All *International Classification of Disease–*based definitions for the baseline and index hospitalization characteristics can be found in the eAppendix in the Supplement.

^b^ At least 1 new cognitive, psychological, or medical diagnosis in the respective time frame.

^c^ Eligibility for long-term care benefits in line with the German Social Code, ranging from grade 1 (ie, “Little impairment of independence”) to grade 5 (“Hardship cases”).

^d^ Total health care costs include cost for hospitalizations, outpatient consultations, medication, treatments (eg, physical or occupational therapy), and rehabilitation. To convert to US dollars, apply the 2017 mean exchange rate of 0.885 €/US $.^26^

**Figure 2.**
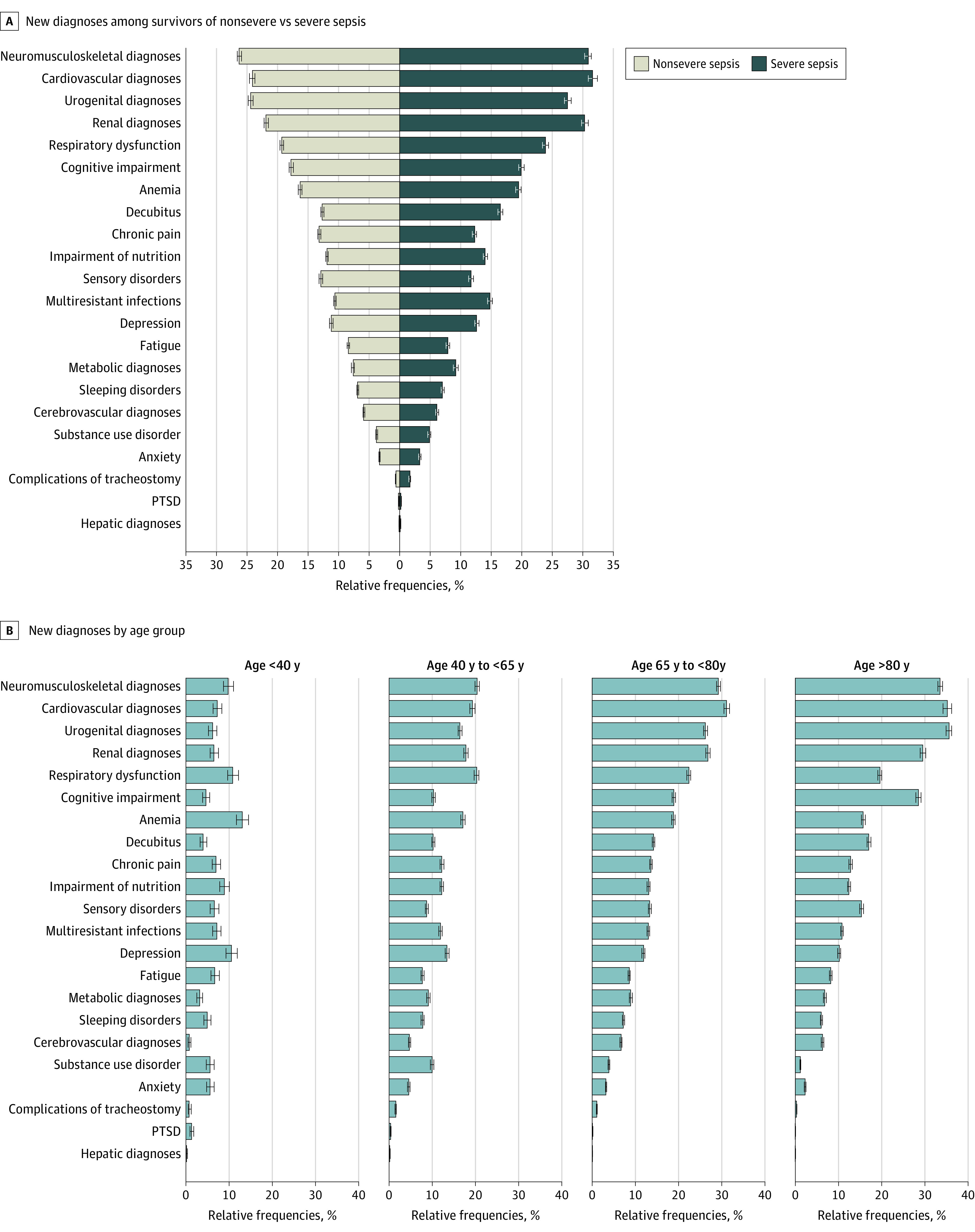
New Postsepsis Diagnoses in the 1 to 12 Months After Hospital Discharge Among Survivors of Nonsevere vs Severe Sepsis and by Age Group PTSD indicates posttraumatic stress disorder.

**Figure 3.**
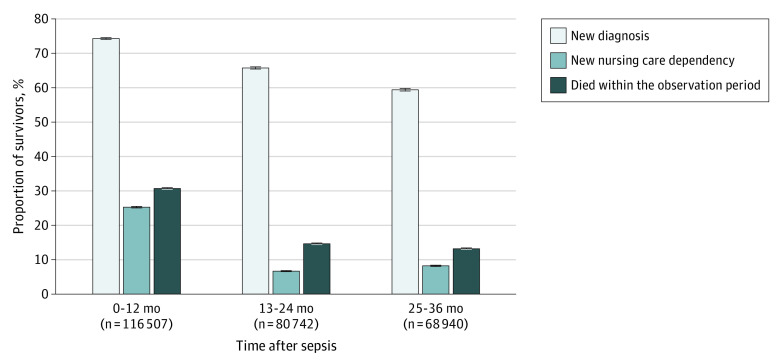
Postsepsis Morbidity and Mortality 1 to 12, 13 to 24, and 25 to 36 Months After Sepsis This figure shows the percentage of afflicted survivors among all sepsis survivors in the first, second, and third year after sepsis. Of note, the proportion of patients with new nursing need is for all sepsis survivors, not just those at risk.

The most common new diagnoses were neuromuscular/musculoskeletal, cardiovascular, respiratory, kidney, and urogenital diseases, occurring in between 21% and 27% of at-risk survivors (Figure 2A; eTable 3 in the Supplement). For example, 12 893 survivors (26.5%; 95% CI, 26.1%-26.9%) were diagnosed with cardiovascular diseases. New diagnoses of decubitus ulcers, chronic pain, and nutritional impairment occurred in 13% to 14% of at-risk survivors. For example, 12 416 survivors (12.9%; 95% CI, 12.7%-13.1%) had a new chronic pain diagnosis. New fatigue occurred in 8925 (8.2%; 95% CI, 8.1%-8.4%), dysphagia in 7572 (6.9%; 95% CI, 6.7%-7.0%), depression in 9878 (11.7%; 95% CI, 11.5%-11.9%), anxiety in 3550 (3.3%; 95% CI, 3.2%-3.4%), and PTSD in 211 (0.2%; 95% CI, 0.2%-0.2%) of at-risk survivors. New diagnoses in 2 and 3 domains affected 23 964 (20.6%; 95% CI, 20.3%-20.8%) and 4441 (3.8%; 95% CI, 3.7%-3.9%), respectively (Figure 1B). In the second and third year post sepsis, new diagnoses occurred in 53 089 (65.8%; 95% CI, 65.4%-66.1%) and 40 959 (59.4%; 95% CI, 59.0%-59.8%) of 1- and 2-year survivors, respectively (Figure 3 and eTable 4 in the Supplement).

### New Diagnoses and Care Dependency by Subgroup

Approximately three-quarters of survivors were older than 65 years. Patients younger than 40 years were less commonly affected by any new diagnosis (56.1%; 95% CI, 54.2%-57.9%) than patients between 40 and 65 years (72.1%; 95% CI, 71.6%-72.7%), those between 65 and 80 years (76.0%; 95% CI, 75.7%-76.4%) or older than 80 years (74.7%; 95% CI, 74.3%-75.2%) (Table 2). Younger patients at risk were also less frequently affected by new dependency on nursing care (6.9%; 95% CI, 5.9%-8.0%) than older patients (between 19.9% and 47.7%). Patients younger than 40 years had fewer medical diagnoses than older patients (48.5% [95% CI, 46.6%-50.4%] of those aged <40 years vs 72.1% [95% CI, 71.6%-72.5%] of those aged >80 years) but comparatively similar rates of depression and anxiety compared with older patients (depression: between 13.4% [95% CI, 13.0%-13.9%] of those aged <40 years and 10.2% [95% CI, 9.8%-10.5%) of those aged >80 years; anxiety: from 5.8% [95% CI, 5.0%-6.8%] of those aged <40 years and 2.3% [95% CI, 2.1%-2.5%] of those aged >80 years) (Figure 2B).

**Table 2.** Postsepsis Morbidity and Mortality at 12 Months, by Age Group and Severity^a^

Outcome	Survivors, % (95% CI)								
	<40 y (n = 2649)	40-65 y (n = 25 860)	66-80 y (n = 51 787)	>80 y (n = 36 211)	Nonsevere sepsis (n = 78 667)^b^	Severe sepsis or septic shock (n = 37 840)^b^	Not treated in ICU (n = 84 269)^b^	Treated in ICU (n = 32 238)^b^	No preexisting diagnosis (n = 8622)
Any new diagnosis^c^	56.1 (54.2-57.9)	72.1 (71.6-72.7)	76.0 (75.7-76.4)	74.7 (74.3-75.2)	73.7 (73.4-74.0)	75.6 (75.1-76.0)	72.8 (72.5-73.1)	78.3 (77.8-78.7)	68.5 (67.5-69.5)
New medical diagnosis	48.5 (46.6-50.4)	67.2 (66.6-67.8)	73.1 (72.7-73.5)	72.1 (71.6-72.5)	70.0 (69.7-70.4)	72.8 (72.3-73.2)	69.3 (69.0-69.6)	75.3 (74.8-75.7)	63.5 (62.4-64.5)
New psychological diagnosis	19.5 (18.1-21.1)	23.0 (22.5-23.5)	18.1 (17.8-18.4)	13.8 (13.5-14.2)	17.4 (17.1-17.6)	19.0 (18.6-19.4)	16.5 (16.3-16.8)	21.4 (21.0-21.9)	25.0 (24.1-25.9)
New cognitive diagnosis^d^	4.8 (4-5.7)	10.3 (9.9-10.7)	18.9 (18.5-19.3)	28.5 (27.9-29.1)	17.8 (17.4-18.1)	19.9 (19.5-20.4)	17.8 (17.4-18.1)	20.2 (19.7-20.7)	12.8 (12.1-13.6)
New diagnoses in 2 domains	14.0 (12.7-15.4)	20.7 (20.2-21.2)	21.2 (20.8-21.5)	20.1 (19.7-20.5)	19.9 (19.6-20.2)	22.0 (21.6-22.4)	19.2 (18.9-19.4)	24.2 (23.7-24.7)	22.1 (21.2-23.0)
New diagnoses in 3 domains	1.2 (0.9-1.7)	3.3 (3.1-3.6)	4.2 (4-4.4)	3.8 (3.6-4)	3.4 (3.3-3.6)	4.6 (4.4-4.8)	3.3 (3.2-3.4)	5.2 (5.0-5.5)	5.4 (4.9-5.9)
New nursing home residence^d^	1.4 (1-2)	6.7 (6.4-7.1)	10.6 (10.4-10.9)	19.6 (19.2-20.1)	11.5 (11.3-11.7)	13.1 (12.7-13.4)	11.5 (11.3-11.8)	13.2 (12.8-13.5)	7.2 (6.7-7.8)
New nursing care^d^^,^^e^	6.9 (5.9-8)	19.9 (19.4-20.5)	32.6 (32.1-33.1)	47.7 (46.9-48.5)	29.9 (29.5-30.3)	34.8 (34.2-35.3)	29.1 (28.7-29.5)	36.8 (36.2-37.5)	19.3 (18.5-20.2)
Mortality	7.9 (6.9-8.9)	21.9 (21.4-22.4)	30.2 (29.8-30.6)	39.4 (38.9-39.9)	29.2 (28.9-29.5)	33.9 (33.4-34.4)	30.3 (30.0-30.6)	31.8 (31.3-32.3)	15.2 (14.4-15.9)
Total health care costs, €^f^									
Mean (SD)	21 847 (49 351)	20 586 (33 973)	15 686 (22 778)	9178 (12 817)	14 372 (24 289)	15 969 (25 610)	13 682 (23 214)	18 051 (28 090)	12 583 (25 932)
Median (IQR)	5391 (803 to 23 102)	9281 (2772 to 24 840)	8049 (2961 to 19 330)	5107 (1797 to 11 565)	6763 (2368 to 16 625)	7736 (2536 to 18 933)	6414 (2223 to 15 814)	9146 (3100 to 21 813)	3716 (812 to 12 696)

Abbreviation: ICU, intensive care unit.

^a^ All *International Classification of Disease–*based definitions for the baseline and index hospitalization characteristics can be found in the eAppendix in the Supplement.

^b^ Differences between groups (nonsevere vs severe sepsis; non-ICU vs ICU) were statistically significant (each *P* < .001).

^c^ At least 1 new cognitive, psychological, or medical diagnosis in the 12 months after index hospitalization discharge.

^d^ New cognitive diagnosis, new nursing home residence, and new nursing care were determined among survivors without these in the 12 months prior to index hospitalization.

^e^ New care level according to German care level system or new nursing home residence, ranging from grade 1 (“Little impairment of independence”) to grade 5 (“Hardship cases”).

^f^ Total health care costs include cost for hospitalizations, outpatient consultations, medication, treatments (eg, physical or occupational therapy), and rehabilitation. To convert to US dollars, apply the 2017 mean exchange rate of 0.885 €/US $.^26^

New-onset diagnoses were more common among survivors of severe vs nonsevere sepsis (any new diagnosis: 75.6% [95% CI, 75.1%-76.0%] vs 73.7% [73.4%-74.0%]; *P* < .001) (Table 2; eTable 5 in the Supplement) and among those treated in the ICU vs those not treated in the ICU (78.3% [95% CI, 77.8%-78.7%] vs 72.8% [72.5%-73.1%]; *P* < .001) (Table 2; eTable 6 in the Supplement). Among survivors with no prior diagnoses, 63.5% (95% CI, 62.4%-64.5%) had a new medical diagnosis, 25.0% (95% CI, 24.1%-25.9%) had a new psychological diagnosis, and 12.8% (95% CI, 12.1%-13.6%) had a new cognitive diagnosis (Table 2; eTable 7 and eFigure 1 in the Supplement).

New nursing care was more common in at-risk survivors of severe vs nonsevere sepsis (34.8% [95% CI, 34.2%-35.3%] vs 29.9% [29.5%-30.3%]; *P* < .001) and in those treated in the ICU vs those not treated in the ICU (36.8% [95% CI, 36.2%-37.5%] vs 29.1% [95% CI, 28.7%-29.5%]; *P* < .001). Overall, 19.3% (95% CI, 18.5%-20.2%) of at-risk survivors without preexisting diseases required new nursing care.

### Long-term Mortality in Sepsis Survivors

One-year postdischarge mortality was 30.7% (35 765 patients; 95% CI, 30.4%-31.0%, and most post–hospital deaths occurred within 100 days of hospital discharge (20 432 deaths [17.6%; 95% CI, 17.4%-17.8%]) (eFigure 2 in the Supplement). One-year post-discharge mortality was higher in patients with severe vs nonsevere sepsis, in those treated in the ICU vs those not treated in the ICU, in patients with vs without preexisting diagnoses, and in older vs younger patients. After approximately 100 to 150 days post discharge, risk of subsequent mortality was similar between patients with severe vs nonsevere sepsis and between those treated in the ICU vs not treated in the ICU. However, mortality remained higher in older patients and patients with preexisting diseases for the full 3 years post sepsis (eFigures 3A, 3B, 3C, 3D, and 3E in the Supplement).

### Total Health Care Costs

Among all sepsis survivors, mean health care costs were €14 891 (US $16 826) per patient (SD, €24 737 [US $27 951]; median, €7055 [US $7972]; IQR, €2422-€17 379 [US $2737-$19 637) in the first year and decreased to a mean of €11 503 (US $12 998) per patient (SD, €20 788 [US $23 489]; median , €5040 [US $5695]; IQR, €1909-€12 813 [US $2157-$14 478]) and a mean of €10 521 (US $11 888) per patient (SD, €19 146 [US $21 634]; median, €4607 [US $5206]; IQR, €1771-€11 573 [US $2001-$13 077]) in the second and third year, respectively (Table 1). Mean total costs were higher for younger patients (€21 847 [US $24 686]; SD, €49 351 [US $55 764]; median, €5391 [US $6092]; IQR, €803-€23 102 [US $907-$26 104]), declined by age group, and were lowest for patients older than 80 years (€9178 [US $10 371]; SD, €12 817 [US $14 482]; median, €5107 [US $5771]; IQR, €1797-€11 565 [US $2031-$13 068) (Table 2). For survivors of severe sepsis compared with survivors of nonsevere sepsis, mean total health care costs were approximately €1600 Euro higher in the first year (Table 2). Similar differences were found in the following years (eTable 5 in the Supplement). Mean costs for patients treated in the ICU were approximately €4400 higher than for those not treated in the ICU in the first year after sepsis (Table 2). This difference was also evident in the second and third year (eTable 6 in the Supplement). Total health care costs for sepsis hospital survivors for 3 years post sepsis were a mean of €29 088 per patient (SD, €44 195; median, €15 903; IQR, €6004-€34 568) or US $32 868 per patient (SD, $49 938; median, $17 968; IQR, $6784-$39 060) when applying a 2017 mean exchange rate of 0.885 €/US $^26^ (eTable 8 in the Supplement).

## Discussion

In this population-based cohort of more than 100 000 survivors of hospital-treated sepsis with longitudinal follow-up to 3 years post discharge, there were high rates of new diagnoses consistent with postsepsis morbidity, new nursing care dependency, and death. Specifically, three-fourths of survivors were diagnosed with a new medical, psychological, or cognitive condition, and one-third died in the first year. Co-occurrence of new diagnoses in more than 1 domain affected one-quarter of survivors. Importantly, and in contrast to many prior studies, this study captured a broad range of sepsis survivors and showed that postsepsis morbidity is not limited to the oldest survivors or those with the most severe illness—but also affects younger survivors and those without preexisting diagnoses.

The rate of new diagnoses consistent with postsepsis morbidity in our cohort may be higher than prior estimates. In a longitudinal cohort of older US residents, sepsis survivors acquired a median of 1 to 2 new functional limitations, and 10.6% developed new moderate to severe cognitive impairment following sepsis.^6^ This prior study suggested—based on the incidence of new functional and cognitive impairment—that sepsis was likely associated with substantial need for new nursing home placement and informal caregiving by family members but was unable to measure these downstream impacts directly. By contrast, our study directly measured the incidence of new nursing care dependency and found that one-third of at-risk sepsis survivors were newly dependent on nursing care, one-fifth had new cognitive diagnoses, and one-eighth of at-risk survivors had a new diagnosis of depression.

With approximately 320 000 patients with sepsis annually in Germany^13^ and an in-hospital mortality rate of 27.0%, the total direct costs for 3-year follow-up health care can be estimated at €6.8 billion (US $7.7 billion) per year. The full economic impact of sepsis would be even higher if one considers the reduced economic productivity of survivors, the need for informal nursing care, and the life-changing effects on caregivers,^27,28^ on whom many survivors depend for physical and financial support. These results highlight the considerable burden of sepsis and its long-lasting and multifaceted sequelae for patients, families, and the health care system.

Although most survivors had new diagnoses consistent with postsepsis morbidity, only 5.5% were discharged to rehabilitation facilities. Cardiovascular diseases were among the most common new diagnoses, likely an important mediator of long-term mortality in sepsis survivors.^29^ The incidence of new pain diagnosis (12.9%) in our cohort is similar to a previous case-control study, which found that 16% of patients treated in the ICU with and without sepsis experienced chronic pain at 6 months, but could detect no difference between the 2 groups.^30^ Fatigue is a severely disabling symptom and an important determinant of quality of life for sepsis survivors.^28^ Fatigue incidence in our study (8.2%) was in a similar range as reported by survivors of hospital-treated COVID-19.^31^ This underscores that fatigue may also be associated with activation of the immune system.^32^ Long-term ventilation is comparatively rare (1.6%) but nonetheless affects several thousand survivors yearly at enormous costs.^33^ The incidence of anxiety (3.3%) or PTSD (0.2%) in our study was much lower than assessed among convenience samples of survivors from a sepsis self-help group (anxiety, 60%; PTSD, 69%).^34^ The difference can be explained because we assessed incident diseases in a population-based cohort. On the other hand, psychological diagnoses may be undercaptured in our cohort if physicians fail to provide a diagnosis for survivor symptoms.^5^

Our study provides new insight into postsepsis morbidity that may also have relevance for survivors of COVID-19. Sepsis is a frequent complication in patients with COVID-19 treated in the ICU and not treated in the ICU.^35^ In a cohort of patients discharged from the hospital, 76% had 1 or more residual symptom at 6 months, including fatigue/muscle weakness (63%), sleep problems (23%), and anxiety or depression (23%).^36^ Furthermore, our findings demonstrate that new medical, psychological, and cognitive diagnoses consistent with postsepsis morbidity are also common among patients who fulfil the criteria of nonsevere sepsis according to the old sepsis 1 and sepsis 2 criteria.^15,16^

These findings raise questions about the sensitivity of the new sepsis 3 definition in terms of the differentiation between patients with uncomplicated infections, which are less likely to cause major long-term morbidity, and patients with severe infections, formerly categorized as sepsis without organ dysfunction. SIRS criteria are no longer part of the current sepsis definition,^37,38^ but the presence of at least 2 SIRS criteria in patients with proven or clinical suspected infection seems to identify an increased risk of major postinfection morbidity and hospital mortality. Overall, our findings highlight the burden of postsepsis morbidity and the need to develop and implement better systems to support survivors.^39^

### Strengths and Limitations

Our study has several strengths. To our knowledge, it is the first study to comprehensively investigate the epidemiology of postsepsis morbidity across a population-based cohort of adult patients of all ages and with different severities of sepsis. The study used nationwide data of the largest health care insurance provider in Germany and covers approximately one-third of all German patients. Our study had a rigorous process to identify the specific diagnoses and diagnostic codes consistent with postsepsis morbidity, based on a multiprofessional panel of experts who care for patients after sepsis.

Several limitations need to be acknowledged. First, the identification of patients with sepsis and their subsequent diagnoses depends on the quality of coding. The explicit sepsis codes we used for case identification may have missed some patients who met clinical criteria for sepsis.^12,40^ Second, measuring postsepsis morbidity based on diagnostic codes may result in misclassification. However, systematic screening of survivors for new medical, psychological, and cognitive diagnoses would not be feasible for such a large, population-based cohort. In Germany, the plausibility of inpatient and outpatient coding is audited by the Medical Services of the Health Care Funds and the Association of Statutory Health Insurance Physicians, which helps ensure the accuracy of coded diagnoses and mitigate the risk of misclassification in this study. Nevertheless, poor awareness of sepsis sequelae among patients and physicians may result in underdiagnosis. Third, our study is observational and cannot establish causality of postsepsis morbidity. However, prior matched studies suggest that sepsis is associated with subsequent morbidity,^9^ particularly functional limitations, cognitive impairment, and select medical conditions. Fourth, our approach only allowed us to identify new-onset diagnoses but not accelerated progression of preexisting diagnoses. Fifth, our data lacked costs of emergency service utilization, transport, therapeutic aid prescriptions, dental care, home care prescription, nursing care, and indirect costs due to productivity loss, so it underestimates the total financial toll of sepsis. Sixth, we also cannot rule out that differences may exist in comparison with the general German patient population, but prior studies have suggested only small differences between AOK and non-AOK beneficiaries in Germany.^41^

## Conclusions

In this study, postsepsis morbidity was common across all age groups and severities of sepsis, and the financial toll of sepsis was substantial. Future research is needed to prevent, screen for, and treat postsepsis morbidity. The development of comprehensive rehabilitation infrastructure also requires a better understanding of the mechanisms of long-term morbidity.

## Notes

Supplement. **eAppendix.** Supplementary Methods and Definitions and Codes for Case Identification **eTable 1.** Patient and Hospitalization Characteristics and Outcomes for 159 684 Index Sepsis Hospitalizations **eTable 2.** Baseline Characteristics of Different Patient Groups **eTable 3.** Underlying Diseases in the Psychological and Medical Domain **eTable 4.** Prevalent and Incident Impairments in Hospital Survivors, 1 to 12, 13 to 24, and 25 to 36 Months After Sepsis **eTable 5.** Comparison of Outcomes and Costs 1 to 12, 13 to 24, and 25 to 36 Months Among Survivors With Nonsevere and Severe Sepsis **eTable 6.** Comparison of Outcomes and Costs 1 to 12, 13 to 24, and 25 to 36 Months Among Patients Treated in the ICU and Those Not Treated in the ICU **eTable 7.** Outcomes and Costs 1 to 12, 13 to 24, and 25 to 36 Months Among Patients Without Preexisting Impairments **eTable 8.** Total Health Care Costs Among Hospital Survivors, 1 to 12, 13 to 24, and 25 to 36 Months After Sepsis **eFigure 1.** Co-occurrence and Mortality in Patients 1 to 12 Months After Discharge From the Index Hospitalization According to Preexisting Impairments **eFigure 2.** Kaplan Meier Survival Curve Until 36 Months After Discharge **eFigure 3.** Hazard Functions for Death for All Patients With Sepsis, Patients With Severe and Nonsevere Sepsis, Patients Treated in the ICU and Not Treated in the ICU, Patients with Sepsis According to Preexisting Impairments and by Age Groups
