# Supplementary material for: Epidemiology and Costs of Postsepsis Morbidity, Nursing Care Dependency, and Mortality in Germany, 2013 to 2017
Source: JAMA Netw Open. 2021 Nov 12;4(11):e2134290. doi: 10.1001/jamanetworkopen.2021.34290 (PMC8590172; doi:10.1001/jamanetworkopen.2021.34290)
Supplement: Supplement. — eAppendix. Supplementary Methods and Definitions and Codes for Case Identification eTable 1. Patient and Hospitalization Characteristics and Outcomes for 159 684 Index Sepsis Hospitalizations eTable 2. Baseline Characteristics of Different Patient Groups eTable 3. Underlying Diseases in the Psychological and Medical Domain eTable 4. Prevalent and Incident Impairments in Hospital Survivors, 1 to 12, 13 to 24, and 25 to 36 Months After Sepsis eTable 5. Comparison of Outcomes and Costs 1 to 12, 13 to 24, and 25 to 36 Months Among Survivors With Nonsevere and Severe Sepsis eTable 6. Comparison of Outcomes and Costs 1 to 12, 13 to 24, and 25 to 36 Months Among Patients Treated in the ICU and Those Not Treated in the ICU eTable 7. Outcomes and Costs 1 to 12, 13 to 24, and 25 to 36 Months Among Patients Without Preexisting Impairments eTable 8. Total Health Care Costs Among Hospital Survivors, 1 to 12, 13 to 24, and 25 to 36 Months After Sepsis eFigure 1. Co-occurrence and Mortality in Patients 1 to 12 Months After Discharge From the Index Hospitalization According to Preexisting Impairments eFigure 2. Kaplan Meier Survival Curve Until 36 Months After Discharge eFigure 3. Hazard Functions for Death for All Patients With Sepsis, Patients With Severe and Nonsevere Sepsis, Patients Treated in the ICU and Not Treated in the ICU, Patients with Sepsis According to Preexisting Impairments and by Age Groups [file jamanetwopen-e2134290-s001.pdf]

Supplemental Online Content

Fleischmann-Struzek C, Rose N, Freytag A, et al. Epidemiology and costs of postsepsis morbidity, nursing care dependency, and mortality in Germany, 2013 to 2017. *JAMA Netw Open.* 2021;4(11):e2134290. doi:10.1001/jamanetworkopen.2021.34290

eAppendix. Supplementary Methods and Definitions and Codes for Case Identification

eTable 1. Patient and Hospitalization Characteristics and Outcomes for 159 684 Index Sepsis Hospitalizations

eTable 2. Baseline Characteristics of Different Patient Groups

eTable 3. Underlying Diseases in the Psychological and Medical Domain

eTable 4. Prevalent and Incident Impairments in Hospital Survivors, 1 to 12, 13 to 24, and 25 to 36 Months After Sepsis

eTable 5. Comparison of Outcomes and Costs 1 to 12, 13 to 24, and 25 to 36 Months Among Survivors With Nonsevere and Severe Sepsis

eTable 6. Comparison of Outcomes and Costs 1 to 12, 13 to 24, and 25 to 36 Months Among Patients Treated in the ICU and Those Not Treated in the ICU

eTable 7. Outcomes and Costs 1 to 12, 13 to 24, and 25 to 36 Months Among Patients Without Preexisting Impairments

eTable 8. Total Health Care Costs Among Hospital Survivors, 1 to 12, 13 to 24, and 25 to 36 Months After Sepsis

eFigure 1. Co-occurrence and Mortality in Patients 1 to 12 Months After Discharge From the Index Hospitalization According to Preexisting Impairments

eFigure 2. Kaplan Meier Survival Curve Until 36 Months After Discharge

eFigure 3. Hazard Functions for Death for All Patients With Sepsis, Patients With Severe and Nonsevere Sepsis, Patients Treated in the ICU and Not Treated in the ICU, Patients with Sepsis According to Preexisting Impairments and by Age Groups

This supplemental material has been provided by the authors to give readers additional information about their work.

© 2021 Fleischmann-Struzek C et al. *JAMA Network Open.*

eAppendix 1. Supplementary Methods and Definitions and Codes for Case Identification

Postsepsis morbidity concept and operationalization

To identify diagnoses associated with postsepsis morbidity, we conducted a comprehensive literature review on reviews, round table/position papers, and large cohort studies investigating impairments following acute care treatment with sepsis or intensive care treatment.[1,9,11,36,39-41] We classified diagnoses as medical, psychological, or cognitive (Table S1). For the identification of each diagnosis in hospital discharge and outpatient data, relevant ICD-10 codes or procedural codes were identified (i) from prior literature,[42-45] or (ii) in the ICD-10-GM and list of procedural codes, to ensure completeness of definitions. The classification and ICD-10-GM definitions were reviewed in an iterative process by experts from the following fields:

- for sepsis rehabilitation Klinik Bavaria, Kreischa (Prof. Oehmichen, Dr. Sauter)
- from the SMOOTH[46] study group (Dr. Konrad Schmidt, A. Freytag)
- from the REPAIR[47] study group (PD Dr. Rosendahl, Dr. Gawlytta)
- for sepsis-related pain (Prof. Meißner) and neurology/geriatrics (PD Preul)
- from the SEPFROK expert panel (Prof. Vollmar (general medicine), Prof. Janssens (intensive care), Dr. Hecker (patient safety), A. Trumann (sepsis survivor), Dr. Rosseau (pulmonology), L. Ullrich (intensive care nursing)).

Table S1: Definition of psychological, cognitive, and medical impairments following sepsis

	**Diagnosis associated with Postsepsis morbidity**		
**Domains**	**Psychological**	**Cognitive**	**Medical**
**Diagnose**	PTSD	Cognitive dysfunction	Respiratory dysfunction
	Depression		Cardiovascular disease<br>- Coronary heart disease and myocardial infarction<br>- Cardiomyopathy<br>- Heart failure<br>- Cardiac arrhythmias
	Anxiety		Cerebrovascular disease
	Sleeping disorders		Renal dysfunction

© 2021 Fleischmann-Struzek C et al. *JAMA Network Open.*

	Substance abuse		Hepatic dysfunction
			Metabolic diseases<br>- Diabetes mellitus<br>- Other metabolic diseases
			Anaemia
			Neuromuscular/musculoskeletal diseases<br>- ICUAW/CIP/CIM<br>- Dysphagia<br>- Voice disorders<br>- Contractures<br>- Immobility
			Decubitus ulcer
			Compl. of tracheostomy<br>- Tracheal stenoses
			Urogenital diseases<br>- Incontinence<br>- Sexual disorders<br>- Urethral stricture
			Sensory disorders<br>- Vestibular disorders<br>- Hearing disorder<br>- Taste and smelling disorders
			Impairment of nutrition
			Chronic pain
			Infection with multi-resistant bacteria
			Fatigue

© 2021 Fleischmann-Struzek C et al. *JAMA Network Open.*

Case identification: Definitions and Codes

The identification of sepsis patients in our study was based on the following ICD-10-GM codes in health claims data. At least one of the codes had to be coded as primary and secondary discharge diagnosis. During the complete observation period, sepsis coding was based on the sepsis-1/2 criteria in Germany. Therefore, severity was classified as: sepsis – all forms; severe sepsis (including septic shock); septic shock; and non-severe sepsis.

Sepsis<br>ICD-10-GM Codes:	
A02.1	Salmonella sepsis
A20.0	Bubonic plague
A20.7	Septicaemic plague
A21.7	Generalized tularaemia
A22.7	Anthrax sepsis
A24.1	Acute or fulminating melioidosis
A26.7	Erysipelothrix sepsis
A28.2	Extraintestinal yersiniosis
A32.7	Listerial sepsis
A39.1	Waterhouse-Friderichsen syndrome
A39.2	Acute meningococcal sepsis
A39.3	Chronic meningogoccal sepsis
A39.4	Meningococcaemia, unspecified
A40	Streptococcal sepsis
A41	Other sepsis
A42.7	Actinomycotic sepsis
A48.3	Toxic shock syndrome
A49.9	Bacterial infection, unspecified
A54.8	Other gonococcal infections
B00.7	Disseminated herpesviral disease
B37.6	Candidal endocarditis
B37.7	Candidal sepsis
B49	Unspecified mycosis
O75.3	Other infection during labour
O85	other puerperal infections
R65.0	Systemic Inflammatory Response Syndrome of infectious origin without organ failure
R65.1	Systemic Inflammatory Response Syndrome of infectious origin with organ failure
R57.2	Septic shock

Severe sepsis<br>ICD-10-GM Codes:	
R65.1	Systemic Inflammatory Response Syndrome of infectious origin with organ failure

© 2021 Fleischmann-Struzek C et al. *JAMA Network Open.*

Septic shock<br>ICD-10-GM Codes:	
R57.2	Septic shock

Characteristics of sepsis and index treatment

Assessed at discharge from the index treatment

**Focus of infection**<br>Respiratory tract<br>ICD-10-GM Codes:	
J01	Acute sinusitis
J02	Acute pharyngitis
J03	Acute tonsillitis
J04	Acute laryngitis and tracheitis
J06	Acute upper respiratory infections of multiple and unspecified sites
J05	Acute obstructive laryngitis [croup] and epiglottitis
J09	Influenza due to identified zoonotic or pandemic influenza virus
J10	Influenza due to identified seasonal influenza virus
J11	Influenza, virus not identified
J12	Viral pneumonia, not elsewhere classified
J13	Pneumonia due to Streptococcus pneumoniae
J14	Pneumonia due to Haemophilus influenzae
J15	Bacterial pneumonia, not elsewhere classified
J16	Pneumonia due to other infectious organisms, not elsewhere classified
J17	Pneumonia in diseases classified elsewhere
J18	Pneumonia, organism unspecified
J20	Acute bronchitis
J21	Acute bronchiolitis
J22	Unspecified acute lower respiratory infection
J44.0	Chronic obstructive pulmonary disease with acute lower respiratory infection
J44.1	Chronic obstructive pulmonary disease with acute exacerbation, unspecified
J86	Pyothorax
J85	Abscess of lung and mediastinum
A15	Respiratory tuberculosis, bacteriologically or histologically confirmed
A16	Respiratory tuberculosis, not confirmed bacteriologically or histologically
U69.00	Hospital-acquired pneumonia in other diseases classified elsewhere
A36	Diphtheria
A37	Whooping cough
B38	Coccidioidomycosis
B39	Histoplasmosis

Abdominal infections<br>ICD-10-GM Codes:	

© 2021 Fleischmann-Struzek C et al. *JAMA Network Open.*

A00	Cholera
A01	Typhoid and paratyphoid fevers
A02	Other salmonella infections
A03	Shigellosis
A04	Other bacterial intestinal infections
A05	Other bacterial foodborne intoxications, not elsewhere classified
A06	Amoebiasis
A07	Other protozoal intestinal diseases
A08	Viral and other specified intestinal infections
A09	Other gastroenteritis and colitis of infectious and unspecified origin
K35	Acute appendicitis
K37	Unspecified appendicitis
K36	Other appendicitis
K57.02	Diverticular disease of small intestine with perforation and abscess without bleeding
K57.03	Diverticular disease of small intestine with perforation and abscess with bleeding
K57.12	Diverticular disease of small intestine without perforation or abscess without bleeding
K57.13	Diverticular disease of small intestine without perforation or abscess wit bleeding
K57.22	Diverticular disease of large intestine with perforation and abscess without bleeding
K57.23	Diverticular disease of large intestine with perforation, abscess and bleeding
K57.32	Diverticular disease of large intestine without perforation or abscess wihout bleeding
K57.33	Diverticular disease of large intestine without perforation or abscess wit bleeding
K57.42	Diverticular disease of both small and large intestine with perforation and abscess without bleeding
K57.43	Diverticular disease of both small and large intestine with perforation, abscess and bleeding
K57.52	Diverticular disease of both small and large intestine without perforation or abscess or bleeding
K57.53	Diverticular disease of both small and large intestine without perforation or abscess with bleeding
K57.82	Diverticular disease of intestine, part unspecified, with perforation and abscess without bleeding
K57.83	Diverticular disease of intestine, part unspecified with perforation, abscess and bleeding
K57.92	Diverticular disease of intestine, part unspecified, without perforation, abscess or bleeding
K57.93	Diverticular disease of intestine, part unspecified, without perforation or abscess with bleeding
K61	Abscess of anal and rectal regions
K65	Peritonitis
K67	Disorders of peritoneum in infectious diseases classified elsewhere
K63.0	Abscess of intestine
K63.1	Perforation of intestine (nontraumatic)

© 2021 Fleischmann-Struzek C et al. *JAMA Network Open.*

K75.0	Abscess of liver
K75.1	Phlebitis of portal vein
K81.0	Cholecystitis
K77.0	Liver disorders in infectious and parasitic diseases classified elsewhere
U69.40!	Recurrent infection due to Clostridium difficile

Wound/soft tissue infection ICD-10-GM Codes:	
A46	Erysipelas
B47	Mycetoma
L03	Phlegmon
L04	Acute lymphadenitis
L08	Other local infections of skin and subcutaneous tissue
L05	Pilonidal cyst
B00	Herpesviral [herpes simplex] infections
B07	Viral warts
B08	Other viral infections characterized by skin and mucous membrane lesions, not elsewhere classified
B09	Unspecified viral infection characterized by skin and mucous membrane lesions
H05.0	Acute inflammation of orbit
H60.2	Malignant otitis externa
H70.0	Acute mastoiditis
J36	Peritonsillar abscess
J39.0	Retropharyngeal and parapharyngeal abscess
J39.1	Other abscess of pharynx
L02	Cutaneous abscess, furuncle and carbuncle

Genitourinary system infection ICD-10-GM Codes:	
N10	Acute tubulo-interstitial nephritis
N15.1	Renal and perinephric abscess
N15.9	Renal tubulo-interstitial disease, unspecified
N34	Urethritis and urethral syndrome
N30	Cystitis
N39.0	Urinary tract infection, site not specified
N41	Inflammatory diseases of prostate
N45	Orchitis and epididymitis
N48.2	Other inflammatory disorders of penis
N49	Inflammatory disorders of male genital organs, not elsewhere classified
N70	Salpingitis and oophoritis
N71	Inflammatory disease of uterus, except cervix
N72	Inflammatory disease of cervix uteri
N73	Other female pelvic inflammatory diseases

© 2021 Fleischmann-Struzek C et al. *JAMA Network Open.*

N74	Female pelvic inflammatory disorders in diseases classified elsewhere
N75	Diseases of Bartholin gland
N76	Other inflammation of vagina and vulva
N77	Vulvovaginal ulceration and inflammation in diseases classified elsewhere
N61	Inflammatory disorders of breast
N98.0	Infection associated with artificial insemination
A59	Trichomoniasis
A55	Chlamydial lymphogranuloma (venereum)
A56	Other sexually transmitted chlamydial diseases

Central nervous system infection ICD-10-GM Codes:	
A39	Meningococcal infection
G00	Bacterial meningitis, not elsewhere classified
G01	Meningitis in bacterial diseases classified elsewhere
G02	Meningitis in other infectious and parasitic diseases classified elsewhere
G03	Meningitis due to other and unspecified causes
G04	Encephalitis, myelitis and encephalomyelitis
G05*	Encephalitis, myelitis and encephalomyelitis in diseases classified elsewhere
G06	Intracranial and intraspinal abscess and granuloma
G07*	Intracranial and intraspinal abscess and granuloma in diseases classified elsewhere
G08	Intracranial and intraspinal phlebitis and thrombophlebitis
A17+	Tuberculosis of nervous system
A81	Atypical virus infections of central nervous system
A83	Mosquito-borne viral encephalitis
A84	Tick-borne viral encephalitis
A85	Other viral encephalitis, not elsewhere classified
A86	Unspecified viral encephalitis
A87	Viral meningitis
A88	Other viral infections of central nervous system, not elsewhere classified
A89	Unspecified viral infection of central nervous system

Cardiovascular system infection ICD-10-GM Codes:	
I32	Pericarditis in diseases classified elsewhere
I33	Acute and subacute endocarditis
I39	Endocarditis and heart valve disorders in diseases classified elsewhere
I40	Acute myocarditis
I41	Myocarditis in diseases classified elsewhere
I80	Thombosis, phlebitis and thrombophlebitis
I38	Endocarditis, valve unspecified
I98.1	Cardiovascular disorders in other infectious and parasitic diseases classified elsewhere

© 2021 Fleischmann-Struzek C et al. *JAMA Network Open.*

Device-related infections ICD-10-GM Codes:	
T82.6	Infection and inflammatory reaction due to cardiac valve prosthesis
T82.7	Infection and inflammatory reaction due to other cardiac and vascular devices, implants and grafts
T83.5	Infection and inflammatory reaction due to prosthetic device, implant and graft in urinary system
T83.6	Infection and inflammatory reaction due to prosthetic device, implant and graft in genital tract
T84.5	Infection and inflammatory reaction due to internal joint prosthesis
T84.6	Infection and inflammatory reaction due to internal fixation device [any site]
T84.7	Infection and inflammatory reaction due to other internal orthopaedic prosthetic devices, implants and grafts
T85.7	Infection and inflammatory reaction due to other internal prosthetic devices, implants and grafts

Pregnancy associated infection ICD-10-GM Codes:	
O75.3	Other infection during labour
O85	Puerperal fever
O03.0	Spontaneous abortion, complicated by genital tract and pelvic infection, incomplete
O03.5	Spontaneous abortion, complicated by genital tract and pelvic infection, complete or unspecified
O04.0	Medical abortion, complicated by genital tract and pelvic infection, incomplete
O04.5	Medical abortion, complicated by genital tract and pelvic infection, complete or unspecified
O05.0	Other abortion, complicated by genital tract and pelvic infectio, incomplete
O05.5	Other abortion, complicated by genital tract and pelvic infection, complete or unspecified
O06.0	Unspecified abortion, complicated by genital tract and pelvic infection, incomplete
O06.5	Unspecified abortion, complicated by genital tract and pelvic infection, complete or unspecified
O07.0	Failed medical abortion, complicated by genital tract and pelvic infection
O07.5	Other and unspecified failed attempted abortion, complicated by genital tract and pelvic infection
O08.0	Genital tract and pelvic infection following abortion and ectopic and molar pregnancy
O86	Other puerperal infections
O23	Infections of genitourinary tract in pregnancy
O41.1	Infection of amniotic sac and membranes
O88.3	Obstetric pyaemic and septic embolism
O91	Infections of breast associated with childbirth

© 2021 Fleischmann-Struzek C et al. *JAMA Network Open.*

O98	Maternal infectious and parasitic diseases classifiable elsewhere but complicating pregnancy, childbirth and the puerperium

Hospital-acquired infections ICD-10-GM Codes:	
T82.6	Infection and inflammatory reaction due to cardiac valve prosthesis
T82.7	Infection and inflammatory reaction due to other cardiac and vascular devices, implants and grafts
T84.5	Infection and inflammatory reaction due to internal joint prosthesis
T84.6	Infection and inflammatory reaction due to internal fixation device [any site]
T84.7	Infection and inflammatory reaction due to other internal orthopaedic prosthetic devices, implants and grafts
T85.72	Infection and inflammatory reaction due to internal prosthetic devices, implants and grafts in the central nervous system
T85.73	Infection and inflammatory reaction due to prosthetic devices or implants of the mamma
T85.75	Infection and inflammatory reaction due to internal prosthetic devices, implants or grafts of the hepatobiliary system or pancreas
T85.76	Infection and inflammatory reaction due to internal prosthetic devices, implants or grafts of the other gastrointestinal system
T85.78	Infection and inflammatory reaction due to other internal prosthetic devices, implants and grafts
O86.0	Infection of obstetric surgical wound
T83.5	Infection and inflammatory reaction due to prosthetic device, implant and graft in urinary system
T83.6	Infection and inflammatory reaction due to prosthetic device, implant and graft in genital tract
A04.7	Enterocolitis due to Clostridium difficile
U69.40!	Recurrent infection due to Clostridium difficile
T80.2	Infections following infusion transfusion and therapeutic injection
T82.7	Infection and inflammatory reaction due to other cardiac and vascular devices, implants and grafts
T81.4	Infection following a procedure, not elsewhere classified
T85.71	Infection and inflammatory reaction due to peritoneal dialysis catheter
T85.74	Infection and inflammatory reaction due to percutaneous endoscopic gastrostomy/jejunostomy,T88.0
U69.00	Hospital-acquired pneumonia in patients aged 18 years or older

Multidrug-resistant infections ICD-10-GM Codes:	
U80.!	Grampositive bacteria with specified antibiotic resistance, requiring special therapeutic or hygienic measures
U81.!	Gram negative bacteria with specified antibiotic resistanc, requiring special therapeutic or hygienic measures
U82.!	Mycobacteria with resistance against TB drugs (first line)
U83.!	Candida with resistance against Fluconazole and Voriconazole
U84.!	Herpes virus with restistance against antivirals

© 2021 Fleischmann-Struzek C et al. *JAMA Network Open.*

U85!	Human Immunodeficiency Virus with resistance against antivirals or proteinase â€“ inhibitors
OPS Codes:	
8-987	Complex treatment in the case of colonisation or infection with multidrug-resistant pathogens [MDR]

**Organ dysfunctions** Cardiovascular dysfunction/shock ICD-10-GM Codes:	
I95.9	Hypotension, unspecified
R57.8	Other shock
R57.9	Shock, unspecified
R57.2	Septic shock

Respiratory dysfunction ICD-10-GM Codes:	
J96	Respiratory failure, not elsewhere classified
J80	Adult respiratory distress syndrome
J98.4	Other disorders of lung
R06.0	Dyspnoea
R06.8	Other and unspecified abnormalities of breathing

Encephalopathy ICD-10-GM Codes:	
F05	Delirium, not induced by alcohol and other psychoactive substances
G93.1	Anoxic brain damage, not elsewhere classified
G93.4	Encephalopathy, unspecified
R40	Somnolence, stupor and coma

Renal dysfunction ICD-10-GM Codes:	
N17.	Acute renal failure
N19	Unspecified kidney failure

Metabolic dysfunction ICD-10-GM Codes:	
E87.2	Acidosis

Abnormal coagulation ICD-10-GM Codes:	
Coagulation D65	Disseminated intravascular coagulation [defibrination syndrome]
D68.8	Other specified coagulation defects

© 2021 Fleischmann-Struzek C et al. *JAMA Network Open.*

D68.9	Coagulation defect, unspecified
D69.5	Secondary thrombocytopenia
D69.6	Thrombocytopenia, unspecified

Hepatic dysfunction ICD-10-GM Codes:	
K72.0	Acute and subacute hepatic failure
K72.7	Hepatic encephalopathy and hepatic coma
K72.9	Hepatic failure, unspecified
K76.2	Central haemorrhagic necrosis of liver
K76.3	Infarction of liver

Other organ dysfunction ICD-10-GM Codes:	
R65.1	Systemic Inflammatory Response Syndrome of infectious origin with organ complications

ICU Treatment OPS Codes:	
8-980	Intensive care complex treatment
8-98f	Costly intensive care complex treatment (basic procedure)
8-98d	Intensive care complex treatment in childhood (basic procedure)
8-98c	Intensive care complex treatment in childhood

Mechanical Ventilation OPS Codes:	
8-713	Mechanical ventilation and respiratory support in adults
8-712	Mechanical ventilation and respiratory support in children and adolescents
8-714	Special procedure for mechanical ventilation in the case of severe respiratory failure
8-70	Access for mechanical ventilation and measures to maintain the airway
8-71	Mechanical ventilation and respiratory support via a mask or tube and ventilation weaning

Renal replacement therapy OPS Codes:	
8-853	Haemofiltration
8-854	Haemodialysis
8-855	Haemodiafiltration
8-857	Peritoneal dialysis
8-85a	Dialysis procedure due to a functional failure and failure of a kidney transplant

© 2021 Fleischmann-Struzek C et al. *JAMA Network Open.*

Tracheostomy during hospitalization<br>OPS Codes:	
5-311	Temporary tracheostomy
5-312	Permanent tracheostomy

Surgical treatment<br>OPS Codes:	
Any OPS Code from Chapter 5	

Amputation during treatment<br>OPS Codes:	
5-862	Amputation and exarticulation of upper extremity
5-863	Amputation and exarticulation of hand
5-864	Amputation and exarticulation of lower extremity
5-865	Amputation and exarticulation of foot
5-866	Revision of amputation area

Palliative care<br>OPS Codes:	
8-982	Palliative medical complex treatment
8-98e	Specialized inpatient palliative medical complex treatment
8-98h	Specialized palliative medical complex treatment through a palliative care service

Early rehabilitation treatment<br>OPS Codes:	
8-55	Interdisciplinary and other early rehabilitation

**Discharge disposition**	**Definition**
regular	regular termination of treatment, with or without post-discharge treatment intended
other hospital	transfer to another hospital;<br>transfer to another hospital as part of a cooperation;<br>external transfer for psychiatric treatment
hospice	discharge into a hospice
rehabilitation	discharge into a rehabilitation facility

© 2021 Fleischmann-Struzek C et al. *JAMA Network Open.*

nursing home	discharge into a long-term care facility
other	treatment terminated for other reasons, with or without post-discharge treatment intended;<br>Treatment terminated against medical advice, with or without post-discharge treatment intended;<br>Change of responsibility of the cost bearer;<br>Death;<br>internal routing;<br>Treatment terminated for other reasons, post-inpatient treatment intended;<br>external transfer with relocation or change between the<br>Remuneration ranges of the DRG flat rate case, according to section 17b (1) first sentence of the Hospital Funding Act;<br>Internal transfer with a change between the DRG fee ranges<br>according to section 17b (1)first sentence of the Hospital Funding Act;<br>Relocation;<br>Discharge before resumption with reclassification;<br>Discharge before resumption with reclassification due to complication;<br>Discharge or transfer with subsequent readmission;<br>Case closure (internal transfer) when changing between full,<br>day-care and ward-equivalent treatment;<br>Start of an outside stay with an absence past midnight<br>(BPflV area - for the specialist department for laying);<br>Ending an outside stay with an absence past midnight<br>(BPflV area - for pseudo specialist department 0003);<br>Discharge at the end of the year if accepted in the previous year (for the purposes of<br>Billing - § 4 PEPPV);<br>Beginning of a period without direct patient contact<br>(station equivalent treatment);<br>Termination of a period without direct patient contact<br>(ward equivalent treatment - for pseudo-specialist department 0004);

12 months prior health and socioeconomic status

© 2021 Fleischmann-Struzek C et al. *JAMA Network Open.*

As data on outpatient diagnoses, cases, case-related therapies and corresponding costs in Germany are available on only a quarterly basis, data from quarters 1-4 prior to hospital admission were included in the 12 months look-back, respectively.
Both primary and secondary hospital discharge diagnoses as well as outpatient diagnoses labeled as confirmed diagnoses by the treating physicians[23] were included in the definition of pre-sepsis health impairments.

**Employment Status**	**Definition**
Employed	Insurance type: 1 = compulsory health insurance, 2 = pension applicant, 5 = self-payer, 6 = rehabilitation
Unemployed	Type of insurance: 3 = pension recipient, 4 = benefits under the Employment Promotion Act, 9 = family insurance

Comorbidities
defined according to Charlson Comorbidity Index[48]

Pre-existing immobility ICD-10-GM Codes:	
R26.2	Difficulty in walking, not elsewhere classified
R26.3	Immobility
R29.6	Tendency to fall, not elsewhere classified
Z99.3	Dependence on wheelchair
Z74.0	Need for assistance due to reduced mobility

Pre-existing long-term mechanical ventilation ICD-10-GM Codes:	
Z99.0	Dependence on aspirator
Z99.1	Dependence on respirator
OPS Codes:	
8-713	Mechanical ventilation and respiratory support in adults
8-712	Mechanical ventilation and respiratory support in children and adolescents
8-714	Special procedure for mechanical ventilation in the case of severe respiratory failure
8-70	Access for mechanical ventilation and measures to maintain the airway
8-71	Mechanical ventilation and respiratory support via a mask or tube and ventilation weaning

© 2021 Fleischmann-Struzek C et al. *JAMA Network Open.*

Pre-existing dialysis ICD-10-GM Codes:	
Z99.2	Dependence on renal dialysis
Z49	Care involving dialysis
OPS Codes:	
5-392	Creation of an arteriovenous fistula
8-853	Haemofiltration
8-854	Haemodialysis
8-855	Haemodiafiltration
8-857	Peritoneal dialysis
Statutory scale of fees for physicians (GOÄ) Codes:	
13602	Flat rate supplementary fee for continuous care of a patient requiring dialysis
13610	Flat rate supplementary fee for medical care in the case of haemodialysis, peritoneal dialysis and special procedures
13611	Flat rate supplementary fee for medical care in the case of peritoneal dialysis
4562	Flat rate supplementary fee for continuous care of a patient requiring dialysis
4564	Flat rate supplementary fee for paediatric nephrology care when carrying out haemodialysis
4565	Flat rate supplementary fee for paediatric nephrology care when carrying out peritoneal dialysis
40815	Flat rate fee for dialysis in patients up to the age of 18 years at their place of residence
40816	Flat rate fee for peritoneal dialysis in patients up to the age of 18 years
40817	Flat rate fee for peritoneal dialysis in patients up to the age of 18 years at their place of residence
40818	Flat rate fee for haemodialysis in patients up to the age of 18 years during a holiday or other absence
40819	Flat rate fee for peritoneal dialysis in patients up to the age of 18 years during a holiday or other absence
40823	Flat rate fee for dialysis in insured persons from the age of 18 years
40824	Flat rate fee for dialysis in insured persons from the age of 18 years at their place of residence
40825	Flat rate fee for peritoneal dialysis in insured persons from the age of 18 years
40826	Flat rate fee for peritoneal dialysis in insured persons from the age of 18 years at their place of residence
40827	Flat rate fee for intermittent peritoneal dialysis in insured persons from the age of 18 years at their place of residence
40828	Flat rate fee for dialysis from the age of 18 years during a holiday or work-related stay
40829	Supplement to flat rate fee 40823 or 40825 for insured persons aged 59-69 years
40830	Supplement to flat rate fee 40824, 40826 and 40827 for insured persons aged 59-69 years
40831	Supplement to flat rate fee 40823 or 40825 for insured persons aged 69-79 years
40832	Supplement to flat rate fee 40824, 40826 and 40827 for insured persons aged 69-79 years

© 2021 Fleischmann-Struzek C et al. *JAMA Network Open.*

40833	Supplement to flat rate fee 40823 or 40825 for insured persons from 79 years of age
40834	Supplement to flat rate fee 40824, 40826 and 40827 for insured persons from 79 years of age
40835	Supplement to flat rate fee 40816, 40823 or 40825 for dialysis in a patient with an infection
40836	Supplement to flat rate fee 40815, 40817, 40818, 40819, 40824, 40826 to 40828 for dialysis in a patient with an infection
40837	Supplement to flat rate fee 40816 or 40825 for intermittent peritoneal dialysis
40838	Supplement to flat rate fee 40817, 40819, 40827 or 40828 for intermittent peritoneal dialysis

Prior organ transplantation ICD-10-GM Codes:	
Z94	Transplanted organ and tissue status
OPS Codes:	
5-504	Liver transplantation
5-375	Heart and heart-lung transplantation
5-555	Kidney transplantation
5-335	Lung transplantation
5-5281	Transplantation of a pancreas segment
5-5282	Transplantation of the pancreas (whole organ)
5-4676	Small intestine transplantation

Prior major surgery OPS Codes:	
5-32	Excision and resection of lung and bronchus
5-33	Other operations on lung and bronchus
5-34	Operations on the chest wall, pleura, mediastinum and diaphragm
5-35	Operations on the valves and septa of the heart and pericardial vessels
5-36	Operations on the coronary vessels
5-37	Surgical treatment of arrhythmias and other operations on the heart and pericardium
8-851	Bypass surgery (using the heart-lung machine)
5-38	Incision excision and occlusion of blood vessels.
5-39	Other operations on blood vessels
5-42	Surgery on the oesophagus
5-43	Incision, excision and resection of the stomach
5-44	Extended stomach resection and other operations on the stomach
5-45	Incision, excision, resection and anastomosis of the small and large intestine
5-46	Other operations on the small and large intestine
5-47	Operations on the appendix
5-48	Operations on the rectum
5-49	Operations on the anus
5-50	Operations on the liver
5-51	Operations on the gallbladder and bile ducts

© 2021 Fleischmann-Struzek C et al. *JAMA Network Open.*

5-52	Operations on the pancreas
5-53	Abdominal hernia repair
5-54	Other operations in the abdominal region
5-78	Operations on other bones
5-79	Reduction of fractures and dislocations
5-80	Open joint surgery
5-81	Arthroscopic joint surgery
5-82	Prosthetic joint and bone replacement
5-83	Operations on the spine
5-84	Operations on the hand
5-85	Operations on muscles, tendons, fasciae and bursae
5-86	Replantation, exarticulation and amputation of extremities and other operations on the organs of locomotion
5-01	Incision (trepanation) and excision of the skull, brain and meninges
5-02	Other operations on the skull, brain and meninges
5-03	Operations on the spinal cord, spinal meninges and spinal canal
5-04	Operations on the nerves and nerve ganglia
5-05	Other operations on the nerves and nerve ganglia
5-55	Operations on the kidneys
5-56	Operations on the ureters
5-57	Operations on the bladder
5-58	Operations on the urethra
5-59	Other operations on the urinary organs
5-60	Operations on the prostate and seminal vesicles
5-61	Operations on the scrotum and tunica vaginalis testis
5-62	Operations on the testicles
5-63	Operations on the spermatic cord, epididymis and vas deferens
5-64	Operations on the penis
5-65	Operations on the ovary
5-66	Operations on the fallopian tubes
5-67	Operations for facial bone fractures
5-68	Incision, excision and removal of the uterus
5-69	Other operations on the uterus and operations on the parametria
5-70	Operations on the vagina and recto-uterine pouch
5-71	Operations on the vulva
5-72	Childbirth with breech presentation and instrumental delivery
5-73	Other operations to induce labour and during the birth
5-74	Caesarean section and child development
5-75	Other obstetric operations
5-87	Excision and resection of the breast
5-88	Other operations on the breast
8-989	Surgical complex treatment in cases of severe infection

Prior palliative treatment<br>ICD-10-GM Codes:	

© 2021 Fleischmann-Struzek C et al. *JAMA Network Open.*

Z51.5	Palliative care
OPS:	
8-982	Palliative medical complex treatment
8-98e	Specialized inpatient palliative medical complex treatment
8-98h	Specialized palliative medical complex treatment through a palliative care service
Statutory scale of fees for physicians (GOÃ„) Codes:	
1425	Initial care in specialized outpatient palliative care
1426	Follow-up prescription for continuation of the specialized outpatient palliative care
3370	Palliative medical initial diagnosis
3371	Supplementary fee for palliative medical care in the medical practice
3372	Supplementary fee for palliative medical care in the home
3373	Supplementary fee for palliative medical care in the home
1425	Initial prescription for specialized outpatient palliative care
1426	Follow-up prescription for continuation of the specialized outpatient palliative care
3370	Palliative medical initial diagnosis of patient status including treatment plan
3371	Supplementary fee to the insured persons flat rate 03000 for palliative medical care of the patient in the medical practice
3372	Supplementary fee to Catalogue of Tariffs for Physicians code 01410 or 01413 for palliative medical care in the home
3373	Supplementary fee to Catalogue of Tariffs for Physicians code 01411, 01412 or 01415 for palliative medical care in the home
4370	Palliative medical initial diagnosis
4371	Supplementary fee to the insured persons flat rate 04000 for palliative medical care of the patient in the medical practice
4372	Supplementary fee to Catalogue of Tariffs for Physicians code 01410 or 01413 for palliative medical care in the home
4373	Supplementary fee to Catalogue of Tariffs for Physicians code 01411, 01412 or 01415 for palliative medical care in the home
37302	Supplementary fee to the insured persons flat rate or basic flat rate for the coordinating panel doctor
37314	Consultation discussion doctor with an additional designation palliative medicine
37318	Telephone consultation
37300	Palliative medical initial diagnosis of patient status including treatment plan
37305	Supplementary fee to tariff codes 01410 and 01413 for palliative medical care in the home
37306	Supplementary fee to tariff codes 01411 01412 and 01415 for palliative medical care in the home
37317	Supplementary fee to tariff code 37302 for accessibility and willingness to visit in critical phases
37320	Case conference

Pre-existing asplenia coded in the five years prior to sepsis index hospitalization	

© 2021 Fleischmann-Struzek C et al. *JAMA Network Open.*

ICD-10-GM Codes:	
Q89.0<br>Q89.01	Asplenia (congenital)<br>Asplenia (congenital)
OPS Codes:	
5-413.1	Splenectomy, total

Postsepsis morbidity

As data on outpatient diagnoses, cases, case-related therapies and corresponding costs in Germany are available on only a quarterly basis, data from quarters 1-4, 5-8 and 9-12 following the quarter of the index hospitalization discharge date were included in the 12, 24 and 36 month follow-up, respectively.

Both primary and secondary hospital discharge diagnoses as well as outpatient diagnoses labeled as confirmed diagnoses by the treating physicians[23] were included in the definition of post-sepsis diagnoses.

Cognitive impairment<br>ICD-10-GM Codes:	
F06.7	Mild cognitive disorder
U51.-	Impairment of cognitive function
R41.0	Disorientation, unspecified
F00*	Dementia in Alzheimer disease
F01	Vascular dementia
F02*	Dementia in other diseases classified elsewhere
F03	Unspecified dementia
F04	Organic amnesic syndrome, not induced by alcohol and other psychoactive substances
F05	Delirium, not induced by alcohol and other psychoactive substances
F06.9	Unspecified organic mental disorder due to brain damage and dysfunction and to physical disease
F07.8	Other organic personality and behavioural disorders due to brain disease, damage and dysfunction
F07.9	Unspecified organic personality and behavioural disorder due to brain disease, damage and dysfunction
G30	Alzheimer disease
G31.0	Circumscribed brain atrophy
G31.1	Senile degeneration of brain, not elsewhere classified
G31.9	Degenerative disease of nervous system, unspecified
G32*	Other degenerative disorders of nervous system in diseases classified elsewhere

Psychological impairment<br>PTSD<br>ICD-10-GM Codes:	
F43	Reaction to severe stress, and adjustment disorders

© 2021 Fleischmann-Struzek C et al. *JAMA Network Open.*

F43.0	Acute stress reaction
F43.1	Post-traumatic stress disorder
F43.2	Adjustment disorders
F43.8	Other reactions to severe stress
F43.9	Reaction to severe stress, unspecified
Depression	
ICD-10-GM Codes:	
F32	Depressive episode
F33	Recurrent depressive disorder
F34.1	Dysthymia
F38	Other mood [affective] disorders
F41.2	Mixed anxiety and depressive disorder
F06.3	Organic mood [affective] disorders
Anxiety	
ICD-10-GM Codes:	
F40	Phobic anxiety disorders
F41	Other anxiety disorders
F06.4	Organic anxiety disorder
Sleeping disorders	
ICD-10-GM Codes:	
F51	Nonorganic sleep disorders
G47	Sleep disorders
Substance abuse	
ICD-10-GM Codes:	
F10	Mental and behavioural disorders due to use of alcohol
F11	Mental and behavioural disorders due to use of opioids
F12	Mental and behavioural disorders due to use of cannabinoids
F13	Mental and behavioural disorders due to use of sedatives or hypnotics
F14	Mental and behavioural disorders due to use of cocaine
F15	Mental and behavioural disorders due to use of other stimulants, including caffeine
F16	Mental and behavioural disorders due to use of hallucinogens
F17	Mental and behavioural disorders due to use of tobacco
F18	Mental and behavioural disorders due to use of volatile solvents
F19	Mental and behavioural disorders due to multiple drug use and use of other psychoactive substances

Medical impairment Respiratory dysfunction ICD-10-GM Codes:	
J96	Respiratory failure, not elsewhere classified
J98	Other respiratory disorders
R06.0	Dyspnoea
J80	Adult respiratory distress syndrome

© 2021 Fleischmann-Struzek C et al. *JAMA Network Open.*

Cardiovascular diseases<br>Coronary heart disease and myocardial infarction<br>ICD-10-GM Codes:	
I20	Angina pectoris
I21	Acute myocardial infarction
I22	Subsequent myocardial infarction
I24	Other acute ischaemic heart diseases
I25	Chronic ischaemic heart disease
Cardiomyopathy	
ICD-10-GM Codes:	
I42	Cardiomyopathy
Heart failure	
ICD-10-GM Codes:	
I50	Heart failure
Cardiac arrhythmias	
ICD-10-GM Codes:	
I47	Paroxysmal tachycardia
I48	Atrial fibrillation and flutter
I49	Other cardiac arrhythmias

Cerebrovascular diseases<br>ICD-10-GM Codes:	
I63	Cerebral infarction
I64	Stroke, not specified as haemorrhage or infarction
I65	Occlusion and stenosis of precerebral arteries, not resulting in cerebral infarction
I66	Occlusion and stenosis of cerebral arteries, not resulting in cerebral infarction

Renal diseases<br>ICD-10-GM Codes:	
N17	Acute renal failure
N18	Chronic kidney disease
N19	Unspecified kidney failure

Hepatic diseases<br>ICD-10-GM Codes:	
K72.1	Chronic hepatic failure

Metabolic diseases<br>Diabetes mellitus<br>ICD-10-GM Codes:	

© 2021 Fleischmann-Struzek C et al. *JAMA Network Open.*

E11	Type 2 diabetes mellitus
E12	Malnutrition-related diabetes mellitus
E13	Other specified diabetes mellitus
E14	Unspecified diabetes mellitus
Other metabolic diseases ICD-10-GM Codes:	
E27	Other disorders of adrenal gland
E35*	Disorders of endocrine glands in diseases classified elsewhere
E34.9	Endocrine disorder, unspecified
E23	Hypofunction and other disorders of pituitary gland

Anaemia ICD-10-GM Codes:	
D50	Iron deficiency anaemia
D51	Vitamin B12 deficiency anaemia
D52	Folate deficiency anaemia
D53	Other nutritional anaemias
D63	Anaemia in chronic diseases classified elsewhere
D64.9	Anaemia, unspecified

Neuromuscular/musculoskeletal diseases ICUAW/CIP/CIM ICD-10-GM Codes:	
G62.8	Critical illness polyneuropathy
G72.8	Critical illness myopathy
Dysphagia	
ICD-10-GM Codes:	
R13	Dysphagia
Voice disorders	
ICD-10-GM Codes:	
R49	Voice disturbances
Contractures	
ICD-10-GM Codes:	
M62.4	Contracture of muscle
M24.5	Contracture of joint
M25.6	Stiffness of joint, not elsewhere classified
M21.62	Acquired Pes equinus
Immobility	
ICD-10-GM Codes:	
R26.2	Difficulty in walking, not elsewhere classified
R26.3	Immobility
R29.6	Tendency to fall, not elsewhere classified
Z99.3	Dependence on wheelchair

© 2021 Fleischmann-Struzek C et al. *JAMA Network Open.*

Z74.0	Need for assistance due to reduced mobility

Decubitus<br>ICD-10-GM Codes:	
L89	Decubitus ulcer and pressure area

Complications of tracheostomy<br>ICD-10-GM Codes:	
Z43.0	Attention to tracheostomy
Z93.0	Tracheostomy status
J95.0	Tracheostomy malfunction

Tracheal stenoses<br>ICD-10-GM Codes:	
J95.5	Postprocedural subglottic stenosis
J95.81	Tracheal stenosis following a procedure
J38.6	Stenosis of larynx
J39.8	Acquired tracheal stenosis

Urogenital diseases<br>Incontinence<br>ICD-10-GM Codes:	
R32	Unspecified urinary incontinence
N39.3	Stress incontinence
N39.4	Other specified urinary incontinence
R15	Faecal incontinence
Sexual disorders	
ICD-10-GM Codes:	
F52	Sexual dysfunction, not caused by organic disorder or disease
Urethral stricture	
ICD-10-GM Codes:	
N99.1	Postprocedural urethral stricture

Sensory disorders<br>Vestibular disorders<br>ICD-10-GM Codes:	
R42	Dizziness and giddiness
Hearing disorder	
ICD-10-GM Codes:	
H90	Conductive and sensorineural hearing loss
H91	Other hearing loss
H93	Other disorders of ear, not elsewhere classified

© 2021 Fleischmann-Struzek C et al. *JAMA Network Open.*

Taste and smelling disorders	
ICD-10-GM Codes:	
R43	Disturbances of smell and taste

Impairment of nutrition<br>ICD-10-GM Codes:	
E41	Nutritional marasmus
E43	Unspecified severe protein-energy malnutrition
E44	Protein-energy malnutrition of moderate and mild degree
E46	Unspecified protein-energy malnutrition
R63.0	Anorexia
R63.3	Feeding difficulties and mismanagement
R63.4	Abnormal weight loss
R63.6	Insufficient intake of food and water
R63.8	Other symptoms and signs concerning food and fluid intake
R64	Cachexia

Multidrug-resistant infections<br>ICD-10-GM Codes:	
U80.-!	Gram-positive bacteria with specified antibiotic resistance requiring special therapeutic or hygienic measures
U81.-!	Gram-negative bacteria with specified antibiotic resistance requiring special therapeutic or hygienic measures
U82	Mycobacteria with resistance against TB drugs (first line)
U83	Candida with resistance against Fluconazole and Voriconazole
U84	Herpes virus with restistance against antivirals

Chronic pain<br>ICD-10-GM Codes:	
R52.1	Chronic intractable pain
R52.2	Other chronic pain
R52.9	Pain unspecified
F45.4	Persistent somatoform pain disorder
F45.41	Chronic pain disorder associated with psychological and behavioural factors
G54.6	Phantom limb syndrome with pain

Fatigue<br>ICD-10-GM Codes:	
R53	Malaise and fatigue
G93.3	Chronic fatigue syndrome

© 2021 Fleischmann-Struzek C et al. *JAMA Network Open.*

Long-term mechanical ventilation<br>ICD-10-GM Codes:	
Z99.0	Dependence on aspirator
Z99.1	Dependence on respirator
OPS Codes:	
8-713	Mechanical ventilation and respiratory support in adults
8-716	Setting up of home mechanical ventilation
8-718	Weaning from mechanical ventilation

Dialysis<br>ICD-10-GM Codes:	
Z99.2	Dependence on renal dialysis
Z49	Care involving dialysis
OPS Codes:	
8-853	Haemofiltration
8-854	Haemodialysis
8-855	Haemodiafiltration
8-857	Peritoneal dialysis
8-85a	Dialysis procedure due to a functional failure and failure of a kidney transplant

© 2021 Fleischmann-Struzek C et al. *JAMA Network Open.*

eTable 1. Patient and Hospitalization Characteristics and Outcomes for 159 684 Index Sepsis Hospitalizations		
**Characteristics and Outcomes**	**Patients, % (95% CI)**	
Age,	mean (SD): 73.8 (12.8)	
	median (IQR): 76 (67, 83)	
Female sex	75809	47.5 (47.2 - 47.7)
Charlson Comorbidity Index,	mean (SD): 2.1 (1.5)	
	median (IQR): 2 (1, 3)	
Admission as emergency	92398	57.9 (57.6 - 58.1)
Site of infection***		
- Respiratory tract	61465	38.5 (38.3 - 38.7)
- Genitourinary system	48877	30.6 (30.4 - 30.8)
- Abdominal	25485	16.0 (15.8 - 16.1)
- Device-related	11601	7.3 (7.1 - 7.4)
- Wound/soft tissue infection	9835	6.2 (6.0 - 6.3)
- Cardiovascular system	5616	3.5 (3.4 - 3.6)
- Central nervous system	1456	0.9 (0.9 - 1.0)
- Pregnancy associated infection	103	0.1 (0.1 - 0.1)
Hospital-acquired infection	33399	20.9 (20.7 - 21.1)
Multi-drug-resistant infection	7780	4.9 (4.8 - 5.0)
Severity of sepsis		
- severe sepsis	69956	43.8 (43.6 - 44.1)
- septic shock	20589	12.9 (12.7 - 13.1)
ICU treatment	54317	34.0 (33.8 - 34.2)
Mechanical ventilation	40129	25.1 (24.9 - 25.3)
Renal replacement therapy	16456	10.3 (10.2 - 10.5)
Tracheostomy during hospitalization	11860	7.4 (7.3 - 7.6)
Surgical treatment (any surgical procedure)	56603	35.4 (35.2 - 35.7)
Amputation during hospitalization	2755	1.7 (1.7 - 1.8)
Palliative care (complex palliative treatment during hospitalization)	2329	1.5 (1.4 - 1.5)
Early rehabilitation treatment (interdisciplinary rehabilitation during hospitalization)	5870	3.7 (3.6 - 3.8)
Hospital length of stay	mean (SD): 20.6 (20.8)	
	median (IQR): 14 (8, 26)	
In-Hospital mortality	43177	27.0 (26.8 - 27.3)
Discharge disposition of survivors		
- home	84482	72.5 (72.3 - 72.8)
- another acute care hospital	14265	12.2 (12.1 - 12.4)
- rehabilitation facility	6397	5.5 (5.4 - 5.6)
- nursing home	9386	8.1 (7.9 - 8.2)
- hospice	310	0.3 (0.2 - 0.3)
- other	1667	1.4 (1.4 - 1.5)
Hospital costs	mean (SD): 15361 (28731); median (IQR): 5849 (3474, 13749)	
**Health status, utilization and costs during the 12 months prior to sepsis**		
Employed	20144	12.6 (12.5 - 12.8)
Dependence on chronic care		
- Nursing home residence	18636	11.7 (11.5 - 11.8)
- Dependency on nursing care according to German graded care system*	61167	38.3 (38.1 - 38.5)
Pre-existing immobility	31657	19.8 (19.6 - 20.0)
Pre-existing long-term mechanical ventilation	2156	1.4 (1.3 - 1.4)
Pre-existing dialysis	6793	4.3 (4.2 - 4.4)
Prior organ transplantation	1412	0.9 (0.8 - 0.9)
Major surgery in the prior year	43772	27.4 (27.2 - 27.6)
Palliative treatment (any inpatient and outpatient palliative care treatment) in the prior year	11683	7.3 (7.2 - 7.4)
Pre-existing asplenia	584	0.4 (0.3 - 0.4)
Total health costs in the year prior to sepsis**	mean (SD): 13074 (19690) median (IQR): 6327 (2126, 15989)	

© 2021 Fleischmann-Struzek C et al. *JAMA Network Open.*

All ICD-based definition for the baseline and index hospitalization characteristics can be found in Supplement 1.
Abbreviations: CCI = Charlson Comorbidity Index, unweighted; IQR = Interquartile range; SD = Standard deviation
* eligibility for long-term care benefits in line with the German Social Code, ranging from Grade 1: “Little impairment of independence” up to Grade 5: “Hardship cases”
** Total health care costs include cost for hospitalizations, outpatient consultations, medication, treatments (e.g. physical or occupational therapy) and rehabilitation.
***Defined by ICD code indicating an infectious source, as indicated in methods supplement. Note that multiple sites of infection were possible.

© 2021 Fleischmann-Struzek C et al. *JAMA Network Open.*

eTable 2. Baseline Characteristics of Different Patient Groups										
	**Non-severe sepsis**		**Severe sepsis**		**ICU-treated sepsis**		**Non-ICU-treated sepsis**		**Sepsis patients w/o pre-existing impairments**	
	**n**	**% (95% CI)**	**n**	**% (95% CI)**	**n**	**% (95% CI)**	**n**	**% (95% CI)**	**n**	**% (95% CI)**
Patients, % of the total cohort	89,728	56.2 (55.9 - 56.4)	69,956	43.8 (43.6 - 44.1)	54,317	34 (33.8 - 34.2)	105,367	66 (65.8 - 66.2)	10,666	6.7 (6.6 - 6.8)
Age: mean (SD); median (IQR)	73.8 (13.2); 76 (67, 83)		73.8 (12.3); 76 (67, 83)		71.6 (12.3); 74 (64, 80)		75 (12.9); 77 (69, 84)		63.1 (16.3); 65 (53, 75)	
Female sex	44,246	49.3 (49 - 49.6)	31,563	45.1 (44.7 - 45.5)	22,504	41.4 (41 - 41.8)	53,305	50.6 (50.3 - 50.9)	4,450	41.7 (40.8 - 42.7)
CCI: mean (SD); median (IQR)	2 (1.4); 2 (1, 3)		2.3 (1.5); 2 (1, 3)		2.4 (1.5); 2 (1, 3)		2 (1.4); 2 (1, 3)		1.2 (1.2); 1 (0, 2)	
Admission as emergency	51,208	57.5 (57.2 - 57.8)	41,185	59.2 (58.8 - 59.5)	3,043	56.6 (56.2 - 57)	61,650	58.5 (58.2 - 58.8)	6,454	61(60 - 61.9)
Focus of infection, %										
- Respiratory tract	27,561	30.7 (30.4 - 31)	33,904	48.5 (48.1 - 48.8)	29,524	54.4 (53.9 - 54.8)	31,941	30.3 (30 - 30.6)	3,954	37.1 (36.2 - 38)
- Abdominal	10,221	11.4 (11.2 - 11.6)	15,264	21.8 (21.5 - 22.1)	13,215	24.3 (24 - 24.7)	12,270	11.6 (11.5 - 11.8)	1,967	18.4 (17.7 - 19.2)
- Wound/soft tissue infection	5,305	5.9 (5.8 - 6.1)	4,530	6.5 (6.3 - 6.7)	3,734	6.9 (6.7 - 7.1)	6,101	5.8 (5.7 - 5.9)	799	7.5 (7 - 8)
- Genitourinary system	28,712	32 (31.7 - 32.3)	20,165	28.8 (28.5 - 29.2)	14,952	27.5 (27.2 - 27.9)	33,925	32.2 (31.9 - 32.5)	2,756	25.8 (25 - 26.7)
- Central nervous system	607	0.7 (0.6 - 0.7)	849	1.2 (1.1 - 1.3)	882	1.6 (1.5 - 1.7)	574	0.5 (0.5 - 0.6)	212	2 (1.7 - 2.3)
- Cardiovascular system	2,814	3.1 (3 - 3.3)	2,802	4 (3.9 - 4.2)	2,529	4.7 (4.5 - 4.8)	3,087	2.9 (2.8 - 3.0)	439	4.1 (3.8 - 4.5)
- Device-related	5,484	6.1 (6 - 6.3)	6,117	8.7 (8.5 - 9)	6,455	11.9 (11.6 -12.2)	5,146	4.9 (4.8 - 5.0)	619	5.8 (5.4 - 6.3)
- Pregnancy associated infection	84	0.1 (0.1 - 0.1)	19	0 (0 - 0)	23	0 (0 - 0-1)	80	0.1 (0.1 - 0.1)	45	0.4 (0.3 - 0.6)
Hospital-acquired infection, %	14,072	15.7 (15.4 - 15.9)	19,327	27.6 (27.3 - 28)	19,089	35.1 (34.7 - 35.5)	14,310	13.6 (13.4 - 13.8)	1,986	18.6 (17.9 - 19.4)
Multi-resistant infection, %	3,242	3.6 (3.5 - 3.7)	4,538	6.5 (6.3 - 6.7)	4,623	8.5 (8.3 - 8.7)	3,157	3 (2.9 - 3.1)	312	2.9 (2.6 - 3.3)
Presence of any acute organ dysfunction*, %	40,175	44.8 (44.4 - 45.1)	62,329	89.1 (88.9 - 89.3)	48,412	89.1 (88.9 - 89.4)	54,092	51.3 (51 - 51.6)	6,246	58.6(57.6 - 59.5)
number of organ dysfunctions: mean (SD); median (IQR)	0.6 (0.8); 0 (0, 1)		2 (1.3); 2 (1, 3)		2.1 (1.4); 2 (1, 3)		0.8 (1); 1 (1, 2)		1.2 (1.4); 2 (0, 2)	
Occurance of septic shock, %	0	0 (0-0)	20,589	29.4 (29.1 - 29.8)	14,244	26.2 (25.9 - 26.6)	6,345	6 (5.9 - 6.2)	1,396	13.1 (12.5 - 13.7)
ICU treatment, %	16,369	18.2 (18 - 18.5)	37,948	54.2 (53.9 - 54.6)	54,317	100 (100-100)	0	0 (0-0)	4,096	38.4 (37.5 - 39.3)
Mechanical ventilation, %	9,368	10.4 (10.2 - 10.6)	30,761	44 (43.6 - 44.3)	32,086	59.1 (58.7 - 59.5)	8,043	7.6 (7.5 - 7.8)	3,037	28.5 (27.6 - 29.3)
Renal replacement therapy, %	3,382	3.8 (3.6 - 3.9)	13,074	18.7 (18.4 - 19)	12,893	23.7 (23.4 - 24.1)	3,563	3.4 (3.3 - 3.5)	937	8.8 (8.3 - 9.3)
Tracheostomy during hospitalization, %	2,857	3.2 (3.1 - 3.3)	9,003	12.9 (12.6 - 13.1)	10,636	19.6 (19.2 - 19.9)	1,224	1.2 (1.1 - 1.2)	1,158	10.9 (10.3 - 11.5)
Surgical treatment, %	24,953	27.8 (27.5 - 28.1)	31,650	45.2 (44.9 - 45.6)	33,093	69.9 (60.5 - 61.3)	23,510	22.3 (22.1 - 22.6)	4,664	43.7 (42.8 - 44.7)
Amputation during treatment, %	1,168	1.3 (1.2 - 1.4)	1,587	2.3 (2.2 - 2.4)	1,470	2.7 (2.6 - 2.8)	1,285	1.2 (1.2 - 1.3)	147	1.4 (1.2 - 1.6)
Palliative care, %	1,454	1.6 (1.5 - 1.7)	875	1.3 (1.2 - 1.3)	530	1.0 (0.9 (1.1)	1,799	1.7 (1.6 - 1.8)	98	0.9 (0.8 - 1.1)
Early rehabilitation treatment, %	3,813	4.2 (4.1 - 4.4)	2,057	2.9 (2.8 - 3.1)	1,644	3 (2.9 - 3.2)	4,226	4 (3.9 - 4.1)	204	1.9 (1.7 - 2.2)
Hospital length of stay: mean (SD); median (IQR)	18.3 (17.7); 13 (8, 22)		23.4 (23.9); 16 (9, 32)		30.4 (27.1); 23 (13, 40)		15.5 (14.2); 11 (8, 19)		21.8 (22.3); 14.0 (8, 28)	
Hospital deaths, mortality, %	11,061	12.3 (12.1 - 12.5)	32,116	45.9 (45.5 - 46.3)	22,079	40.6 (40.2 - 41.1)	21,098	20 (19.8 - 20.3)	2,044	19.2 (18.4 - 19.9)
Discharge disposition of survivors, %										
- regular discharge	60,940	77.5 (77.2 - 77.7)	23,542	62.2 (61.7 - 62.7)	18,227	56.5 (56 - 57.1)	66,255	78.6 (78.3 - 78.9)	6,441	60.4 (59.5 - 61.3)
- other hospital	7,079	9 (8.7 - 9.3)	7,186	19 (18.5 - 19.5)	7,112	22.1 (21.6 - 22.5)	7,153	8.5 (8.3 - 8.7)	1,195	11.2 (10.3 - 12.2)
- rehabilitation	3,092	3.9 (3.6 - 4.2)	3,305	8.7 (8.2 - 9.2)	4,097	12.7 (12.3 - 13.1)	2,300	2.7 (2.6 - 2.8)	546	5.1 (4.2 - 6.1)

© 2021 Fleischmann-Struzek C et al. *JAMA Network Open.*

eTable 2. Baseline Characteristics of Different Patient Groups										
	**Non-severe sepsis**		**Severe sepsis**		**ICU-treated sepsis**		**Non-ICU-treated sepsis**		**Sepsis patients w/o pre-existing impairments**	
	**n**	**% (95% CI)**	**n**	**% (95% CI)**	**n**	**% (95% CI)**	**n**	**% (95% CI)**	**n**	**% (95% CI)**
- nursing home	6,120	7.8 (7.5 - 8.1)	3,266	8.6 (8.1 - 9.1)	2,276	7.1 (6.8 - 7.3)	7,110	8.4 (8.3 - 8.6)	252	2.4 (1.4 - 3.3)
- hospice	222	0.3 (0 - 0.6)	88	0.2 (0 - 0.7)	86	0.3 (0.2 - 0.3)	224	0.3 (0.2- 0.3)	16	0.2 (0 - 1.1)
- other	1,214	1.5 (1.3 - 1.8)	453	1.2 (0.7 - 1.7)	440	1.4 (1.2 - 1.5)	1,227	1.5 (1.4 - 1.5)	2,216	20.8 (19.8 - 21.7)
Hospital costs: mean (SD); median (IQR)	9,797 (18,446); 4,410 (3,357, 8,562)		22,496 (36,840); 9,281 (4,410, 27,334)		31,656 (42,027); 17,587 (7,619, 39,944)		6,960 (11,534); 3,928 (3,300, 6,947)		18,232 (30,578); 6,086 (3,442, 19,792)	
**Health status and health care use 12 months prior to index hospitalization**										
Employed persons, %	11,865	13.2 (13 - 13.4)	8,279	11.8 (11.6 - 12.1)	7,554	13.9 (13.6-14.2)	12,590	11.9 (11.8 -12.1)	3,638	34.1 (33.2 - 35)
Dependence on chronic care										
- Nursing home residence, %	10,591	11.8 (11.6 - 12)	8,045	11.5 (11.3 - 11.7)	4,058	4.5 (7.3 - 7.7)	14,578	13.8 (13.6 - 14.0)	96	0.9 (0.7 - 1.1)
- Care level per German care level system**, %	34,364	38.3 (38 - 38.6)	26,803	38.3 (38 - 38.7)	16,951	31.2 (30.8 - 31.6)	44,216	42 (41.7 - 42.3)	626	5.9 (5.4 - 6.3)
Pre-existing immobility, %	17,490	19.5 (19.2 - 19.8)	14,167	20.3 (20 - 20.6)	9,184	16.9 (16.6 - 17.2)	22,473	21.3 (21.1 - 21.6)	0	0 (0 - 0)
Pre-existing long-term mechanical ventilation, %	1,024	1.1 (1.1 - 1.2)	1,132	1.6 (1.5 - 1.7)	1,073	2.0 (1.9 - 2.1)	1,083	1 (1-1.1)	0	0 (0 - 0)
Pre-existing dialysis, %	3,453	3.8 (3.7 - 4)	3,340	4.8 (4.6 - 4.9)	2,865	5.3 (5.1 - 5.5)	3,928	3.7 (3.6 - 3.8)	3	0 (0 - 0.1)
Prior organ transplantation, %	704	0.8 (0.7 - 0.8)	708	1 (0.9 - 1.1)	580	1.1 (1.0 - 1.2)	832	0.8 (0.7 - 0.8)	25	0.2 (0.2 - 0.3)
Prior major surgery, %	24,786	27.6 (27.3 - 27.9)	18,986	27.1 (26.8 - 27.5)	14,791	27.2 (26.9 - 27.6)	28,981	27.5 (27.2 - 27.8)	992	9.3 (8.8 - 9.9)
Prior palliative treatment, %	6,637	7.4 (7.2 - 7.6)	5,046	7.2 (7 - 7.4)	2,945	5.4 (5.2 - 5.6)	8,738	8.3 (8.1 - 8.5)	252	2.4 (2.1 - 2.7)
Pre-existing asplenia, coded in the five years prior to sepsis index hospitalization, %	343	0.4 (0.3 - 0.4)	241	0.3 (0.3 - 0.4)	206	0.4 (0.3 - 0.4)	378	0.4 (0.3 - 0.4)	12	0.1 (0.1 - 0.2)
Total health care costs [€]***, mean (SD); median (IQR)	12,982 (19,255); 6,194 (2,066, 15,891)		13,192 (20,233); 6,495 (2,203, 16,114)		13,344 (21,175); 6,141 (1,992, 15,995)		12,935 (18,877); 6,423 (2,194, 15,985)		3,089 (9,001); 443 (70, 1,869)	

IQR = Interquartile range; SD = Standard deviation
* any ICD-10 code for organ dysfunction, not necessarily linked to infection/sepsis, as the coding of organ dysfunction in sepsis patients is not mandatory in Germany
** eligibility for long-term care benefits in line with the German Social Code
*** Total health care costs include cost for hospitalizations, outpatient consultations, medication and treatments (e.g. physical or occupational therapy) and rehabilitation.

© 2021 Fleischmann-Struzek C et al. *JAMA Network Open.*

eTable 3. Underlying Diseases in the Psychological and Medical Domain						
	Follow-Up after index admission					
	1-12 months		13-24 months		25-36 months	
	n	% (95 % CI)	n	% (95 % CI)	n	% (95 % CI)
**Sepsis survivors, n**	**116,507**		**80,742**		**68,940**	
**1. Medical Diagnoses**						
° Prevalence of physical diagnoses						
°° respiratory dysfunction	31,595	27.1 (26.9 - 27.4)	19,514	24.2 (23.9 - 24.5)	16,247	23.6 (23.3 - 23. 9)
°° cardiovascular diseases	69,406	59.6 (59.3 - 59.9)	49,877	61.8 (61.4 - 62.1)	42,413	61.5 (61.2 - 61.9)
°°° coronary heart disease and myocardial infarction	41,838	35.9 (35.6 - 36.2)	30,724	38.1 (37.7 - 38.4)	25,929	37.6 (37.3 - 38.0)
°°° cardiomyopathy	4,949	4.2 (4.1 - 4.4)	3,523	4.4 (4.2 - 4.5)	3,004	4.4. (4.2 -4.5)
°°° heart failure	43,178	37.1 (36.8 - 37.3)	29,714	36.8 (36.5 - 37.1)	25,423	36.9 (36.5 - 37.2)
°°° cardiac arrhythmias	40,166	34.5 (34.2 - 34.7)	28,349	35.1 (34.8 - 35.4)	24,120	35.0 (34.6 - 35.3)
°° cerebrovascular diseases	16,361	14.0 (13.8 - 14.2)	12,016	14.9 (14.6 - 15.1)	10,185	14.8 (14.5 - 15.0)
°° renal diseases	49,446	42.4 (42.2 - 42.7)	34,692	43.0 (42.6 - 43.3)	30,068	43.6 (43.2 - 44)
°° hepatic diseases	155	0.1 (0.1 - 0.2)	80	01. (0.1 - 01.)	59	01. (0.1 - 0.1)
°° metabolic diseases	51,027	43.8 (43.5 - 44.1)	37,984	47.0 (46.7 - 47.4)	32,452	47.1 (46.7 - 47.4)
°°° diabetes mellitus	50,369	43.2 (42.9 - 43.5)	37,475	46.4 (46.1 - 46.8)	32,033	46.5 (46.1 - 46.8)
°°° other metabolic diseases	1,278	1.1 (1.0 - 1.2)	978	1.2 (1.1 - 1.3)	852	1.2 (1.2 - 1.3)
°° anaemia	30,701	26.4 (26.1 - 26.6)	19,044	23.6 (23.3 - 23.9)	15,913	23.1 (22.8 - 23.4)
°° neuromuscular/musculoskeletal diseases	40,962	35.2 (34.9 - 35.4)	27,069	33.5 (33.2 - 33.9)	23,333	33.8 (33.5 - 34.2)
°°° ICUAW/CIP/CIM	2,659	2.3 (2.2 -2.4)	1,334	1.7 (1.6 -1.7)	1,103	1.6 (1.5 - 1.7)
°°° dysphagia	10,285	8.8 (8.7 - 9.0)	5,751	7.1 (6.9 - 7.3)	4,688	6.8 (6.6 - 7.0)
°°° voice disorders	1,337	1.1 (1.1 -1.2)	956	1.2 (1.1 - 1.3)	818	1.2 (1.1 - 1.3)
°°° contractures	2,732	2.3 (2.3 - 2.4)	1,893	2.3 (2.2 - 2.5)	1,508	2.2. (2.1 - 2.3)
°°° immobility	33,707	28.9 (28.7 - 29.2)	22,594	28.0 (27.7 - 28.3)	19.688	28.6 (28.2 - 28.9)
°° decubitus	19,774	17.0 (16.8 - 17.2)	9,839	12.2 (12.0 - 12.4)	7,885	11.4 (11.2 - 11.7)
°° complications of the tracheostoma	954	0.8 (0.8 - 0.9)	385	0.5 (0.4 - 0.5)	222	0.3 (0.3 - 0.4)
°°° tracheal stenoses	448	0.4(0.4 - 0.4)	275	0.3 (0.3 -0.4)	186	0.3 (0.2 - 0.3)
°° urogenital diseases	45,951	39.4 (39.2 - 39.7)	30,952	38.3 (38.0 - 38.7)	26,060	37.8 (37.4 - 38.2)
°°° incontinence	44,396	38.1 (37.8 - 38.4)	29,607	36.7 (36.3 - 37.0)	24,865	36.1 (35.7 -36.4)
°°° sexual disorders	2,203	1.9 (1.8 - 2.0)	1,841	2.3 (2.2 - 2.4)	1,639	2.4 (2.3 - 2.5)
°°° urethral stricture	215	0.2 (0.2 - 0.2)	146	0.2 (0.2 - 0.2)	118	0.2 (0.1 - 0.2)
°° sensory disorders	26,224	22.5 (22.3 - 22.7)	20,473	25.4 (25.1 - 25.7)	17,931	26.0 (25.7 - 26.3)
°°° vestibular disorders	11,377	9.8 (9.6 - 9.9)	9,130	11.3 (11.1 -11.5)	8,189	11.9 (11.6 - 12.1)
°°° hearing disorder	18,003	15.5 (15.2 - 15.7)	13,941	17.3 (17.0 - 17.5)	12,035	17.5 (17.2 - 17.7)
°°° taste and smelling disorders	220	0.2 (0.2 - 0.2)	161	0.2 (0.2 - 0.2)	138	0.2 (0.2 - 0.2)
°° impairment of nutrition	17,835	15.3 (15.1 - 15.5)	8,710	10.8 (10.6 - 11.0)	6,705	9.7 (9.5- 9.9)
°° chronic pain	24,833	21.3 (21.1 - 21.6)	20,167	25.0 (24.7 - 25.3)	19,499	28.3 (27.9 - 28.6)
°° multi-resistant infection	15,792	13.6 (13.4 - 13.8)	7,402	9.2 (9.0 - 9.4)	5,419	7.9 (7.7 -8.1)
°° fatigue	11,179	9.6 (9.4 - 9.8)	6,529	8.1 (7.9 - 8.3)	5,375	7.8 (7.6 - 8.0)
° Incidence of new medical diagnoses, n, % of population at risk						
°° respiratory dysfunction	18,899	20.7 (20.5 - 21.0)	9,022	14.7 (14.4 -15.0)	7,704	14.1 (13.8 - 14.4)
°° cardiovascular diseases	12,893	26.5 (26.1 - 26.9)	4,228	14.0 (13.6 - 14.4)	3,800	14.0 (13.6 - 14.4)
°°° coronary heart disease and myocardial infarction	7,647	10.5 (10.3 - 10.7)	3,161	6.4 (6.2 - 6.6)	2,623	6.1 (5.9 - 6.3)
°°° cardiomyopathy	1,992	1.8 (1.7 - 1.9)	775	1.0 (0.9 - 1.1)	653	1.0 (0.9 - 1.1)
°°° heart failure	15,064	19.5 (19.2 - 19.8)	5,967	11.8 (11.5 - 12.1)	5,515	12.3 (12.0 - 12.6)
°°° cardiac arrhythmias	12,389	15.4 (15.2 - 15.7)	4,106	7.9 (7.7 - 8.1)	3,580	7.8 (7.6 - 8.1)
°° cerebrovascular diseases	5,927	5.9 (5.8 - 6.1)	2,704	4.0 (3.8 - 4.1)	2,295	3.4 (3.7 - 4.1)
°° renal diseases	18,801	24.5 (24.2 - 24.8)	6,710	14.4 (14.0 - 14.7)	5,814	14.2 (13.8 - 14.5)
°° hepatic diseases	120	0.1 (0.1 - 0.1)	48	0.1 (0.0 - 0.1)	30	0.0 (0.0 - 0.1)
°° metabolic diseases	5,109	8.1 (7.9 - 8.3)	2,060	4.9 (4.7 - 5.1)	1,732	4.7 (4.5 - 5)
°°° diabetes mellitus	4,859	7.6 (7.4 - 7.8)	1,935	4.5 (4.3 - 4.7)	1,638	4.4 (4.2 -2.6)
°°° other metabolic diseases	597	0.5 (0.5 - 0.6)	301	0.4 (0.3 - 0.4)	259	0.4 (0.3 - 0.4)
°° anaemia	15,937	17.3 (17.1 -17.6)	5,474	9.2 (9.0 - 9.4)	4,730	8.8 (8.6 - 9.1)
°° neuromuscular/ musculoskeletal diseases	24,756	27.8 (27.5 - 28.1)	8,562	16.1 (15.8 - 16.4)	7,613	16.0 (15.6 - 16.3)
°°° ICUAW/CIP/CIM	2,470	2.1 (2.0 - 2.2)	273	0.3 (0.3 - 0.4)	232	0.3 (0.3 - 0.4)
°°° dysphagia	7,572	6.9 (6.7 - 7.0)	2,625	3.5 (3.4 - 3.6)	2,277	3.5 (3.4 - 3.7)
°°° voice disorders	983	0.9 (0.8 - 0.9)	587	0.7 (0.7 - 0.8)	495	0.7 (0.7 - 0.8)
°°° contractures	1,982	1.7 (1.7 - 1.8)	1,009	1.3 (1.2 - 1.4)	774	1.1 (1.1 - 1.2)
°°° immobility	21,653	22.8 (22.5 - 35.1)	7,990	13.8 (13.5 - 14.0)	6,950	13.5 (13.3 - 13.8)

© 2021 Fleischmann-Struzek C et al. *JAMA Network Open.*

eTable 3. Underlying Diseases in the Psychological and Medical Domain						
	**Follow-Up after index admission**					
	**1-12 months**		**13-24 months**		**25-36 months**	
	**n**	**% (95 % CI)**	**n**	**% (95 % CI)**	**n**	**% (95 % CI)**
°° decubitus	14,832	13.9 (13.7 - 14.1)	4,460	6.4 (6.2 - 6.6)	3,891	6.3 (6.1 - 6.5)
°° complications of the tracheostoma	859	0.7 (0.7 - 0.8)	182	0.2 (0.2 -0.3)	97	0.1 (0.1 -0.2)
°°° tracheal stenoses	362	0.3 (0.3 - 0.3)	129	0.2 (0.1 - 0.2)	68	0.1 (0.1 - 0.1)
°° urogenital diseases	20,510	25.4 (25.1 - 15.7)	6,688	13.6 (13.3 -13.9)	5,728	13.0 (12.7 - 13.3)
°°° incontinence	20,480	24.7 (24.4 - 25.0)	6,498	12.8 (12.5 - 13.1)	5,566	12.3 (12.0 - 12.6)
°°° sexual disorders	614	0.5 (0.5 - 0.6)	428	0.5 (0.5 - 0.6)	359	0.5 (0.5 - 0.6)
°°° urethral stricture	164	0.1 (0.1 - 0.2)	83	0.1 (0.1 - 0.1)	63	0.1 (0.1 - 0.1)
°° sensory disorders	10,879	12.5 (12.3 - 12.7)	6,234	10.6 (10.4 -10.9)	5,370	10.6 (10.4 - 10.9)
°°° vestibular disorders	6,627	6.4 (6.2 - 6.5)	3,682	5.2 (5.0 - 5.4)	3,117	5.1 (5.0 - 5.3)
°°° hearing disorder	7,197	7.5 (7.4 - 7.7)	4,455	6.8 (6.6 - 7.0)	3,861	6.8 (6.6 - 7.1)
°°° taste and smelling disorders	137	0.1 (0.1 - 0.1)	80	0.1 (0.1 - 0.1)	65	0.1 (0.1 - 0.1)
°° impairment of nutrition	13,251	12.6 (12.4 - 12.8)	4,853	6.8 (6.6 - 7.0)	4,000	6.3 (6.1 -6.5)
°° chronic pain	12,416	12.9 (12.7 - 13.1)	6,586	10.5 (10.3 - 10.8)	5,671	10.9 (10.7 - 11.2)
°° Multi-resistant infections	13,189	12.0 (11.8 - 12.2)	3,835	5.4 (5.2 - 5.6)	3,077	4.8 (4.7 - 5.0)
°° fatigue	8,925	8.2 (8.1 - 8.4)	4,305	5.8 (5.7 - 6.0)	3,604	5.6 (5.4 - 5.8)
**2. Psychological Diagnoses**						
° Prevalence of psychological diagnoses						
°° PTSD	431	0.4 (0.3 - 0.4)	377	0.5 (0.4 - 0.5)	368	0.5 (0.5 - 0.6)
°° Depression	31,953	27.4 (27.2 - 27.7)	24,550	30.4 (30.1 - 30.7)	21,367	31.0 (30.6 - 31.3)
°° Anxiety	8,192	7.0 (6.9 - 7.2)	6,506	9.1 (7.9 - 8.2)	5,653	8.2 (8.0 - 8.4)
°° Sleeping disorders	17,058	14.6 (14.4 - 14.8)	12,923	16 (15.8 - 16.3)	11,180	16.2 (15.9 - 16.5)
°° Substance abuse	15,142	13.0 (12.8 - 13.2)	11,358	14.1 (13.8 - 14.3)	9,876	14.3 (14.1 - 14.6)
° Incidence of new psychological diagnoses, n, % of population at risk						
°° PTSD	211	0.2 (0.2 - 0.2)	121	0.2 (0.1 - 0.2)	109	0.2 (0.1 - 0.2)
°° Depression	9,878	11.7 (11.5 - 11.9)	4,276	7.7 (7.5 - 7.9)	3,388	7.1 (6.9 - 7.4)
°° Anxiety	3,550	3.3 (3.2 - 3.4)	1,983	2.7 (2.6 - 2.8)	1,585	2.5 (2.4 - 2.6)
°° Sleeping disorders	6,833	6.9 (6.8 - 7.1)	3,569	5.3 (5.2 - 5.5)	2,880	5.0 (4.8 - 5.2)
°° Substance abuse	4,103	4.1 (4.0 - 4.2)	1,868	2.7 (2.6 - 2.8)	1,635	2.8 (2.6 - 2.9)

© 2021 Fleischmann-Struzek C et al. *JAMA Network Open.*

eTable 4. Prevalent and Incident Impairments in Hospital Survivors, 1 to 12, 13 to 24, and 25 to 36 Months After Sepsis

	Follow-Up after index admission					
	**1-12 months**		**13-24 months**		**25-36 months**	
**Survivors, n**	**116507**	**% (95% CI)**	**80742**	**% (95% CI)**	**68940**	**% (95% CI)**
**1. Medical impairment**						
- Prevalence of medical impairment, n, % of survivors	102,018	87.6 (87.4 - 87.8)	73,828	91.4 (91.2 - 91.6)	62,896	91.2 (91 - 91.4)
- New onset of medical impairments, n, % of survivors	82,629	70.9 (70.7 - 71.2)	49,486	61.3 (61 - 61.6)	37,885	55.0 (54.6 - 55.3)
- Number of new onset diseases, mean (SD); median (IQR)	1.9 (1.9); 1 (0, 3)		1.4 (1.6); 1 (0, 2)		1.1 (1.4); 1 (0, 2)	
- Prevalence of mechanical ventilation, n, % of sepsis survivors	2,607	2.2 (2.2 - 2.3)	1,841	2.3 (2.2 - 2.4)	1,605	2.3 (2.2 - 2.4)
- Incidence of mechanical ventilation, n, % of population at risk	1,890	1.6 (1.6 - 1.7)	906	1.1 (1.1 - 1.2)	751	1.1 (1 - 1.2)
- Prevalence of dialysis, n, % of sepsis survivors	6,527	5.6 (5.5 - 5.7)	4,195	5.2 (5 - 5.4)	3,433	5 (4.8 - 5.1)
- Incidence of dialysis, n, % of population at risk	3,144	2.8 (2.7 - 2.9)	1,040	1.4 (1.3 - 1.4)	789	1.2 (1.1 - 1.3)
**2. Psychological impairment**						
- Prevalence of psychological impairment, n, % of survivors	50,950	43.7 (43.4 - 44)	38,598	47.8 (47.5 - 48.1)	33,431	48.5 (48.1 - 48.9)
- New onset of psychological impairment, n, % of survivors	20,840	17.9 (17.7 - 18.1)	10,296	12.8 (12.5 - 13)	8,429	12.2 (12 - 12.5)
- Number of new onset diseases, mean (SD); median (IQR)	1.2 (0.5); 1 (1, 1)		1.2 (0.4); 1 (1, 1)		1.1 (0.4); 1 (1, 1)	
**3. Cognitive impairment**						
- Prevalence of cognitive impairment, n, % of survivors	37,275	32 (31.7 - 32.3)	25,352	31.4 (31.1 - 31.7)	21,425	31.1 (30.7 - 31.4)
- Incidence of cognitive impairment , n, % of survivors at risk	15,955	18.5 (18.2 - 18.7)	5,383	9.8 (9.5 - 10)	4,807	9.8 (9.6 - 10.1)
**4. Dependency on chronic care**						
- Nursing home residence, %	22,468	19.3 (19.1 - 19.5)	15,149	18.8 (18.5 - 19.0)	12,676	18.4 (18.1 - 18.7)
- Care level according to German care level system*, %	58,209	50.0 (49.7 - 50.2)	40,936	50.7 (50.4 - 51.0)	35,577	51.6 (51.2 - 52.0)
- New nursing home residence, % survivors at risk	12,485	12.0 (11.8 - 12.2)	2,223	3.3 (3.2 - 3.5)	1,950	3.4 (3.3 - 3.5)
- New care level according to German care level system*, % survivors at risk	23,572	31.5 (31.1 - 31.8)	3,784	9.2 (9.0 - 9.5)	4,272	11.8 (11.5 - 12.1)
**5. Total health care costs**						
Total health care costs [€]**, mean (SD); median (IQR)	14,891 (24,737); 7,055 (2,422, 17,379)		11,503 (20,788); 5,040 (1,909, 12,813)		10,521 (19,146); 4,607 (1,771, 11,573)	

IQR = Interquartile range; SD = Standard deviation
* eligibility for long-term care benefits in line with the German Social Code
**Total health care costs include cost for hospitalizations, outpatient consultations, medication and treatments (e.g. physical or occupational therapy) and rehabilitation.

© 2021 Fleischmann-Struzek C et al. *JAMA Network Open.*

eTable 5. Comparison of Outcomes and Costs 1 to 12, 13 to 24, and 25 to 36 Months Among Survivors With Nonsevere and Severe Sepsis						
	**Follow- up**					
	**1-12 months**		**13-24 months**		**25-36 months**	
		**% (95% CI)**		**% (95% CI)**		**% (95% CI)**
**1. Medical impairment**						
- Prevalence of medical impairment, n, % of survivors						
Non-severe sepsis	6,9311	88.1 (87.9 - 88.3)	50,582	90.8 (90.5 - 91)	43,157	90.6 (90.3 - 90.9)
Severe sepsis	32,707	86.4 (86.1 - 86.8)	23,246	92.9 (92.6 - 93.2)	19,739	92.6 (92.3 - 93)
p-value	< 0.001		< 0.001		< 0.001	
- New onset of medical impairments, n, % of survivors						
Non-severe sepsis	55,094	70 (69.7 - 70.4)	33,918	60.9 (60.5 - 61.3)	26,142	54.9 (54.4 - 55.3)
Severe sepsis	27,535	72.8 (72.3 - 73.2)	15,568	62.2 (61.6 - 62.8)	11,743	55.1 (54.4 - 55.8)
p-value	< 0.001		< 0.001		0.616	
**2. Psychological impairment**						
- Prevalence of psychological impairment, n, % of survivors						
Non-severe sepsis	34,278	43.6 (43.2 - 43.9)	26,185	47 (46.6 - 47.4)	22,684	47.6 (47.2 - 48.1)
Severe sepsis	16,672	44.1 (43.6 - 44.6)	12,413	49.6 (49 - 50.2)	10,747	50.4 (49.8 - 51.1)
p-value	0.119		< 0.001		< 0.001	
- New onset of psychological impairments, n, % of survivors						
Non-severe sepsis	13,665	17.4 (17.1 - 17.6)	8,394	15.1 (14.8 - 15.4)	5,715	12 (11.7 - 12.3)
Severe sepsis	7,175	19 (18.6 - 19.4)	3,911	15.6 (15.2 - 16.1)	2,714	12.7 (12.3 - 13.2)
p-value	< 0.001		0.039		0.007	
**3. Cognitive impairment**						
- Prevalence of cognitive impairment, n, % of survivors						
Non-severe sepsis	25,316	32.2 (31.9 - 32.5)	17,627	31.6 (31.2 - 32)	14,892	31.3 (30.9 - 31.7)
Severe sepsis	11,959	31.6 (31.1 - 32.1)	7,725	30.9 (30.3 - 31.5)	6,533	30.7 (30 - 31.3)
p-value	0.049		0.032		0.109	
- Incidence of cognitive impairment, n, % of population at risk						
Non-severe sepsis	10,278	17.8 (17.4 - 18.1)	3,721	9.7 (9.4 - 10)	3,271	9.7 (9.4 - 10)
Severe sepsis	5,677	19.9 (19.5 - 20.4)	1,662	9.8 (9.4 - 10.3)	1,536	10.1 (9.6 - 10.6)
p-value	< 0.001		0.779		0.192	
**4.Co-occurence of cognitive/psychological and medical impairments**						
- Prevalent impairments in two domains, n, % of survivors						
Non-severe sepsis	31,398	39.9 (39.6 - 40.3)	23,401	42 (41.6 - 42.4)	20,038	42.1 (41.6 - 42.5)
Severe sepsis	15,095	39.9 (39.4 - 40.4)	10,833	43.3 (42.7 - 43.9)	9,256	43.4 (42.8 - 44.1)
p-value	0.951		0.001		0.001	
- Prevalent impairments in all three domains, n, % of survivors						
Non-severe sepsis	13,279	16.9 (16.6 - 17.1)	9,457	17 (16.7 - 17.3)	8,105	17 (16.7 - 17.4)
Severe sepsis	6,497	17.2 (16.8 - 17.6)	4,375	17.5 (17 - 18)	3,777	17.7 (17.2 - 18.2)
p-value	0.221		0.075		0.024	
- New onset of impairments in two domains, n, % of survivors						
Non-severe sepsis	15,631	19.9 (19.6 - 20.2)	8,315	14.9 (14.6 - 15.2)	5,591	11.7 (11.5 - 12)
Severe sepsis	8,333	22 (21.6 - 22.4)	3,945	15.8 (15.3 - 16.2)	2,677	12.6 (12.1 - 13)
p-value	< 0.001		0.002		0.002	
- New onset of impairments in all three domains, n, % of survivors						
Non-severe sepsis	2,711	3.4 (3.3 - 3.6)	1,077	1.9 (1.8 - 2.1)	636	1.3 (1.2 - 1.4)
Severe sepsis	1,730	4.6 (4.4 - 4.8)	481	1.9 (1.8 - 2.1)	311	1.5 (1.3 - 1.6)
p-value	< 0.001		0.943		0.209	
**5. Dependence on chronic care**						
- Nursing home residence, %						
Non-severe sepsis	15,013	19.1 (18.8 - 19.4)	10,330	18.5 (18.2 - 18.9)	8,654	18.2 (17.8 - 18.5)
Severe sepsis	7,455	19.7 (19.3 - 20.1)	4,819	19.3 (18.8 - 19.8)	4,022	18.9 (18.4 - 19.4)
p-value	0.013		0.015		0.029	
- Care level according to German care level system*, %						
Non-severe sepsis	39,206	49.8 (49.5 - 50.2)	27,760	49.8 (49.4 - 50.2)	24,181	50.8 (50.3 - 51.2)
Severe sepsis	19,003	50.2 (49.7 - 50.7)	13,176	52.7 (52 - 53.3)	11,396	53.5 (52.8 - 54.1)
p-value	0.225		< 0.001		< 0.001	
- Incident nursing home residence, %						
Non-severe sepsis	8,060	11.5 (11.3 - 11.7)	1,573	3.4 (3.2 - 3.6)	1,386	3.5 (3.3 - 3.7)
Severe sepsis	4,425	13.1 (12.7 - 13.4)	650	3.2 (3 - 3.4)	564	3.2 (3 - 3.5)
p-value	< 0.001		0.148		0.096	
- New care level according to German care level system*, %						
Non-severe sepsis	14,931	29.9 (29.5 - 30.3)	4,000	13.1 (12.8 - 13.5)	3,019	11.9 (11.5 - 12.3)
Severe sepsis	8,641	34.8 (34.2 - 35.3)	1,645	13 (12.4 - 13.6)	1,253	11.7 (11.1 - 12.3)
p-value	< 0.001		0.674		0.736	

© 2021 Fleischmann-Struzek C et al. *JAMA Network Open.*

eTable 5. Comparison of Outcomes and Costs 1 to 12, 13 to 24, and 25 to 36 Months Among Survivors With Nonsevere and Severe Sepsis						
	**Follow- up**					
	**1-12 months**		**13-24 months**		**25-36 months**	
		**% (95% CI)**		**% (95% CI)**		**% (95% CI)**
**6. Health care costs**						
- Total health care costs**, mean (SD)						
Non-severe sepsis	14,372 (24,289)		11,057 (20,579)		10,205 (19,339)	
Severe sepsis	15,969 (25,610)		12,498 (21,213)		11,226 (18,687)	
p-value	<0.001		<0.001		<0.001	

SD = Standard deviation
* eligibility for long-term care benefits in line with the German Social Code
** Total health care costs include cost for hospitalizations, outpatient consultations, medication and treatments (e.g. physical or occupational therapy) and rehabilitation.

© 2021 Fleischmann-Struzek C et al. *JAMA Network Open.*

eTable 6. Comparison of Outcomes and Costs 1 to 12, 13 to 24, and 25 to 36 Months Among Patients Treated in the ICU and Those Not Treated in the ICU						
	**Follow-up**					
	**1-12 months**		**13-24 months**		**25-36 months**	
	**n**	**% (95% CI)**	**n**	**% (95% CI)**	**n**	**% (95% CI)**
**1. Medical diagnosis**						
- Prevalence of medical diagnosis, n, % of survivors						
Non-ICU-treated sepsis	73,907	87.7 (87.5 - 87.9)	53,558	91.1 (90.9 - 91.4)	45,560	90.9 (90.6 - 91.1)
ICU-treated sepsis	28,111	87.2 (86.8 - 87.6)	20,270	92.2 (91.9 - 92.6)	17,336	92.2 (91.8 - 92.6)
p-value	0.019		< 0.001		< 0.001	
- New onset of medical diagnosis, n, % of survivors						
Non-ICU-treated sepsis	58,369	69.3 (69 - 69.6)	35,984	61.2 (60.8 - 61.6)	27,688	55.2 (54.8 - 55.7)
ICU-treated sepsis	24,260	75.3 (74.8 - 75.7)	13,502	61.4 (60.8 - 62.1)	10,197	54.2 (53.5 - 55)
p-value	< 0.001		0.624		0.022	
**2. Psychological diagnosis**						
- Prevalence of psychological diagnosis, n, % of survivors						
Non-ICU-treated sepsis	35,915	42.6 (42.3 - 43.0)	27,240	46.4 (46.0 - 46.8)	23,596	47.1 (46.6 - 47.5)
ICU-treated sepsis	15,035	46.6 (46.1 - 47.2)	11,358	51.7 (51.0 - 52.3)	9,835	52.3 (51.6 - 53.0)
p-value	< 0.001		< 0.001		< 0.001	
- New onset of psychological diagnosis, n, % of survivors						
Non-ICU-treated sepsis	13,937	16.5 (16.3 - 16.8)	7,309	12.4 (12.2 - 12.7)	5,989	11.9 (11.7 - 12.2)
ICU-treated sepsis	6,903	21.4 (21.0 - 21.9)	2,987	13.6 (13.1 - 14.0)	2,440	13.0 (12.5 - 13.5)
p-value	< 0.001		< 0.001		< 0.001	
**3. Cognitive diagnosis**						
- Prevalence of cognitive diagnosis, n, % of survivors						
Non-ICU-treated sepsis	28,011	33.2 (32.9 - 33.6)	19,329	32.9 (32.5 - 33.3)	16,203	32.3 (31.9 - 32.7)
ICU-treated sepsis	9,264	28.7 (28.2 - 29.2)	6,023	27.4 (26.8 - 28.0)	5,222	27.8 (27.1 - 28.4)
p-value	< 0.001		< 0.001		< 0.001	
- Incidence of cognitive diagnosis , n, % of population at risk						
Non-ICU-treated sepsis	10,728	17.8 (17.4 - 18.1)	3,938	9.9 (9.7 - 10.2)	3,451	9.9 (9.6 - 10.2)
ICU-treated sepsis	5,227	20.2 (19.7 - 20.7)	1,445	9.3 (8.8 - 9.8)	1,356	9.7 (9.3 - 10.2)
p-value	< 0.001		0.019		664	
**4.Co-occurence of cognitive/psychological and medical diagnoses**						
- Prevalent diagnoses in two domains, n, % of survivors						
Non-ICU-treated sepsis	33,300	39.5 (39.2 - 39.8)	24,539	41.8 (41.4 - 42.2)	20,969	41.8 (41.4 - 42.3)
ICU-treated sepsis	13,193	40.9 (40.4 - 41.5)	9,695	44.1 (43.5 - 44.8)	8,325	44.3 (43.6 - 45.0)
p-value	< 0.001		< 0.001		< 0.001	
- Prevalent diagnoses in all three domains, n, % of survivors						
Non-ICU-treated sepsis	14,509	17.2 (17.0 - 17.5)	10,276	12.2 (12.0 - 12.4)	8,766	10.4 (10.2 - 10.6)
ICU-treated sepsis	5,267	16.3 (15.9 - 16.7)	3,556	11.0 (10.7 - 11.4)	3,116	9.7 (9.3 - 10.0)
p-value	< 0.001		< 0.001		0.005	
- New onset of diagnoses in two domains, n, % of survivors						
Non-ICU-treated sepsis	16,162	19.2 (18.9 - 19.4)	8,765	14.9 (14.6 - 15.2)	5,922	11.8 (11.5 - 12.1)
ICU-treated sepsis	7,802	24.2 (23.7 - 24.7)	3,495	15.9 (15.4 - 16.4)	2,346	12.5 (12 - 13)
p-value	< 0.001		0.001		0.017	
- New onset of diagnoses in all three domains, n, % of survivors						
Non-ICU-treated sepsis	2,763	3.3 (3.2 - 3.4)	1,096	1.9 (1.8 - 2)	673	1.3 (1.2 - 1.4)
ICU-treated sepsis	1,678	5.2 (5 - 5.5)	462	2.1 (1.9 - 2.3)	274	1.5 (1.3 - 1.6)
p-value	< 0.001		0.032		0.262	
**5. Dependence on nursing care**						
- Nursing home residence, %						
Non-ICU-treated sepsis	16,840	20.0 (19.7 - 20.3)	11,496	19.6 (19.2 - 19.9)	9,617	19.2 (18.8 - 19.5)
ICU-treated sepsis	5,628	17.5 (17.0 - 17.9)	3,653	16.6 (16.1 - 17.1)	3,059	16.3 (15.8 - 16.8)
p-value	< 0.001		< 0.001		< 0.001	
- Care level according to German care level system*, %						
Non-ICU-treated sepsis	42,305	50.2 (49.9 - 50.5)	29,704	50.5 (50.1 - 51.0)	25,780	51.4 (51.0 - 51.9)
ICU-treated sepsis	15,904	49.3 (48.8 - 49.9)	11,232	51.1 (50.4 - 51.8)	9,797	52.1 (51.4 - 52.8)
p-value	0.008		< 0.001		< 0.001	
- Incident home residence, %						
Non-ICU-treated sepsis	8,535	11.5 (11.3 - 11.8)	1,703	3.5 (3.4 - 3.7)	1,502	3.6 (3.4 - 3.8)
ICU-treated sepsis	3,950	13.2 (12.8 - 13.5)	520	2.8 (2.6 - 3.1)	448	2.8 (2.6 – 3.1)
p-value	< 0.001		< 0.001		< 0.001	
- Incident care level according to German care level system*, %						
Non-ICU-treated sepsis	15,041	29.1 (28.7 - 29.5)	2,797	9.3 (9.0 - 9.6)	3,190	12.0 (11.7 - 12.4)
ICU-treated sepsis	8,531	36.8 (36.2 - 37.5)	987	9.1 (8.6 – 9.7)	1,082	11.2 (10.6 – 11.8)
p-value	< 0.001		0.577		0.023	
**6. Health care costs**						
- Total health care costs**, mean (SD)						
Non-ICU-treated sepsis	13,682 (23,214)		10,831 (19,764)		10,020 (17,905)	
ICU-treated sepsis	18,051 (28,090)		13,300 (23,213)		11,857 (22,062)	
p-value	< 0.001		< 0.001		< 0.001	
SD = Standard deviation; * eligibility for long-term care benefits in line with the German Social Code; **Total health care costs include cost for hospitalizations, outpatient consultations, medication and treatments (e.g. physical or occupational therapy) and rehabilition.						

© 2021 Fleischmann-Struzek C et al. *JAMA Network Open.*

eTable 7. Outcomes and Costs 1 to 12, 13 to 24, and 25 to 36 Months Among Patients Without Preexisting Impairments						
	**Follow-up**					
	**1-12 months**		**13-24 months**		**25-36 months**	
	**n**	**% (95% CI)**	**n**	**% (95% CI)**	**n**	**% (95% CI)**
**Survivors, n**	8,622		7,314		6,869	
**1. Medical diagnosis**						
- Prevalence of medical diagnosis, n, % of survivors	5,472	63.5 (62.4 - 64.5)	4,382	59.9 (58.8 - 61)	4,236	61.7 (60.5 - 62.8)
- New onset of medical diagnosiss, n, % of survivors	5,472	63.5 (62.4 - 64.5)	2,942	40.2 (39.1 - 41.4)	2,437	35.5 (34.4 - 36.6)
**2. Psychological diagnosis**						
- Prevalence of psychological diagnosis, n, % of survivors	2,157	25 (24.1 - 25.9)	1,820	24.9 (23.9 - 25.9)	1,790	26.1 (25 - 27.1)
- New onset of psychological diagnosis, n, % of survivors	2,157	25 (24.1 - 25.9)	880	12 (11.3 - 12.8)	685	10 (9.3 - 10.7)
**3. Cognitive diagnosis**						
- Prevalence of cognitive diagnosis, n, % of survivors	1,106	12.8 (12.1 - 13.6)	834	11.4 (10.7 - 12.2)	818	11.9 (11.2 - 12.7)
- Incidence of cognitive diagnosis, n, % of survivors at risk	1,106	12.8 (12.1 - 13.6)	327	4.5 (4.0 - 5.0)	244	3.6 (3.1 - 4.0)
**4.Co-occurence of cognitive/psychological and medical diagnosis**						
- Prevalent diagnoses in two domains, n, % of survivors	1,905	22.1 (21.2 - 23)	1,543	21.1 (20.2 - 22)	1,501	21.9 (20.9 - 22.8)
- Prevalent diagnoses in all three domains, n, % of survivors	462	5.4 (4.9 - 5.9)	320	4.4 (3.9 - 4.9)	331	4.8 (4.3 - 5.4)
- New onset of diagnoses in two domains, n, % of survivors	1,905	22.1 (21.2 - 23)	679	9.3 (8.6 - 10)	526	7.7 (7.1 - 8.3)
- New onset of diagnoses in all three domains, n, % of survivors	462	5.4 (4.9 - 5.9)	65	0.9 (0.7 - 1.1)	41	0.6 (0.4 - 0.8)
**5. Dependency on chronic care**						
- Nursing home residence, %	663	7.7 (7.1 - 8.3)	466	6.4 (5.8 - 7)	459	6.7 (6.1 - 7.3)
- Care level according to German care level system*, %	1,979	23 (22.1 - 23.9)	1,498	20.5 (19.6 - 21.4)	1,489	21.7 (20.7 - 22.7)
- New nursing home residence, % survivors at risk	615	7.2 (6.7 - 7.8)	91	1.3 (1.1 - 1.6)	88	1.4 (1.1 - 1.7)
- New care level according to German care level system*, % survivors at risk	1,582	19.3 (18.5 - 20.2)	216	3.7 (3.2 - 4.2)	283	5.1 (4.5 - 5.7)
**6. Total health care costs**						
Total health care costs [€]**, mean (SD); median (IQR)	12,583 (25,932); 3,716 (812, 12,696)		7,004 (20,972); 1,486 (481, 5,741)		5,621 (14,215); 1,312 (469, 4,569)	

IQR=interquartile range; SD=standard deviation; * eligibility for long-term care benefits in line with the German Social Code; ** Total health care costs include cost for hospitalizations, outpatient consultations, medication and treatments (e.g. physical or occupational therapy) and rehabilitation.

© 2021 Fleischmann-Struzek C et al. *JAMA Network Open.*

eTable 8: Total health care costs hospital survivors, 1-12, 13-24 and 25-36 months after sepsis			
	**Sepsis**	**Severe Sepsis**	**Non-Severe Sepsis**
	n; mean (SD); median (IQR)		
12 months prior to index	116,507; 12,451 (18,767); 5,893 (1,954, 15,074)	37,840; 12,243 (18,833); 5,891 (1,954, 14,723)	78,667; 12,550 (18,734); 5,895 (1,954, 15,278)
Index hospitalization	116,507; 13,601 (26,281); 5,514 (3,471, 11,027)	37,840; 22,636 (36,943); 8,752 (4,673, 26,152)	78,667; 9,255 (17,559); 4,185 (3,362, 8,043)
12 months after index	116,507; 14,891 (24,737); 7,055 (2,422, 17,379)	37,840; 15,969 (25,610); 7,736 (2,536, 18,933)	78,667; 14,372 (24,289); 6,763 (2,368, 16,625)
24 months after index	80,742; 11,503 (20,788); 5,040 (1,909, 12,813)	25,020; 12,498 (21,213); 5,638 (2,138, 14,194)	55,722; 11,057 (20,579); 4,784 (1,823, 12,176)
36 months after index	68,940; 10,521 (19,146); 4,607 (1,771, 11,573)	21,313; 11,226 (18,687); 5,058 (1,943, 12,789)	47,627; 10,205 (19,339); 4,419 (1,706, 11,049)
Total costs 0-36 months follow up*	116,507; 29,088 (44,195); 15,903 (6,004, 34,568)	37,840; 30,555 (43,815); 16,893 (5,866, 37,452)	78,667; 28,383 (44,359; 15,505 (6,069, 33,206)
IQR = Interquartile range; SD = Standard deviation; *Total health care costs include cost for hospitalizations, outpatient consultations, medication and treatments (e.g. physical or occupational therapy) and rehabilitation.			

© 2021 Fleischmann-Struzek C et al. *JAMA Network Open.*

eFigure 1. Co-occurrence and Mortality in Patients 1 to 12 Months After Discharge From the Index Hospitalization According to Preexisting Impairments

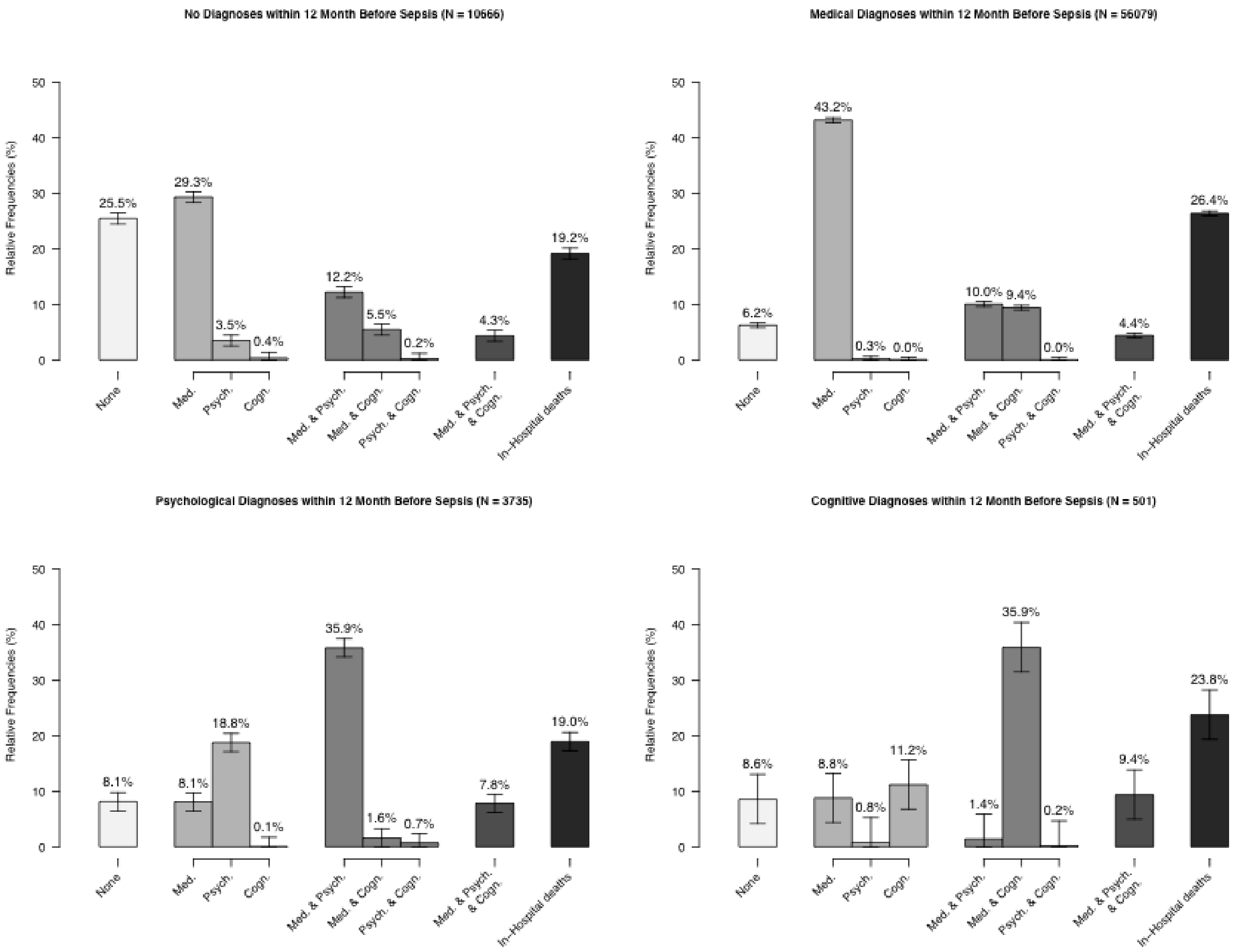

© 2021 Fleischmann-Struzek C et al. *JAMA Network Open.*

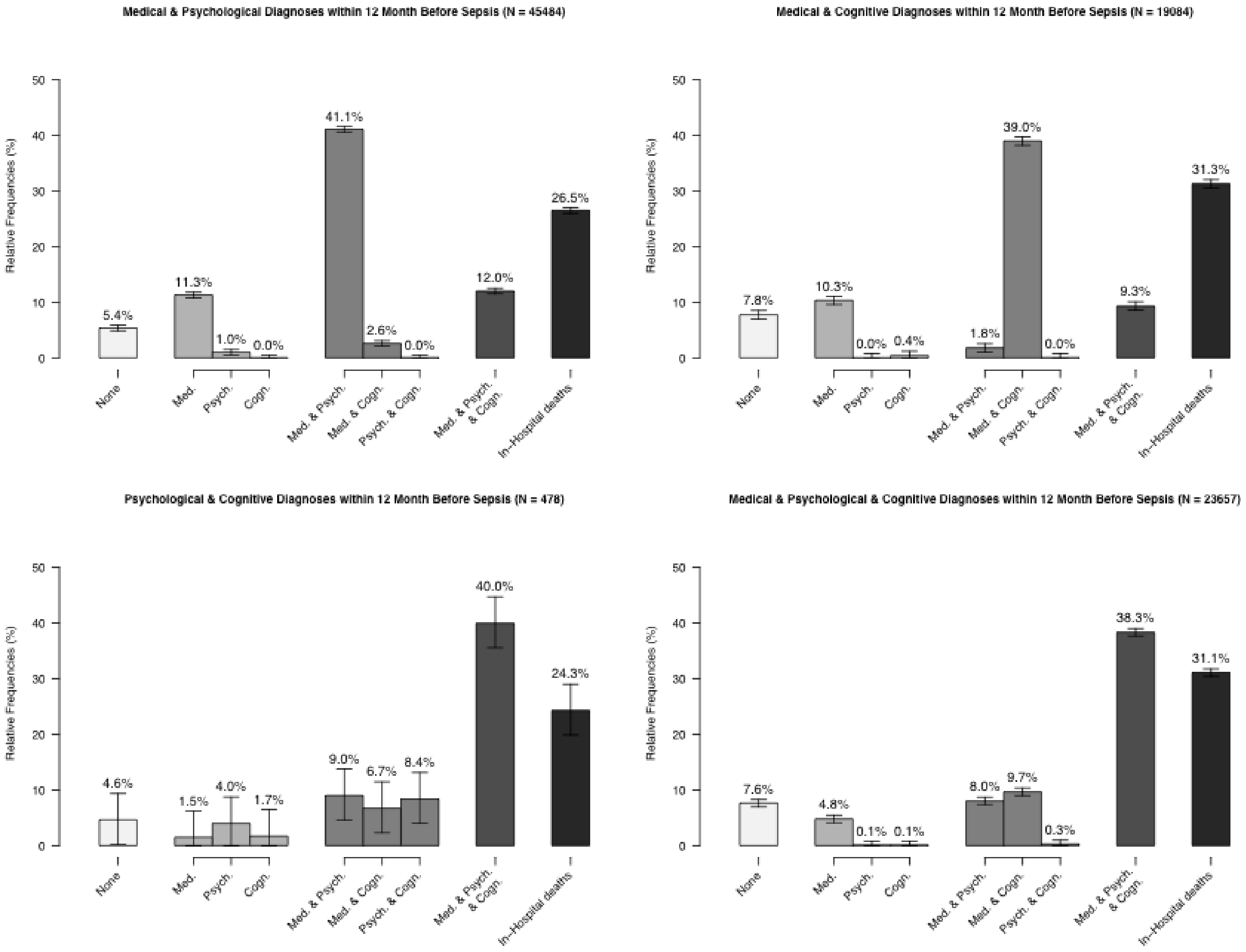

© 2021 Fleischmann-Struzek C et al. *JAMA Network Open.*

eFigure 2. Kaplan Meier Survival Curve Until 36 Months After Discharge

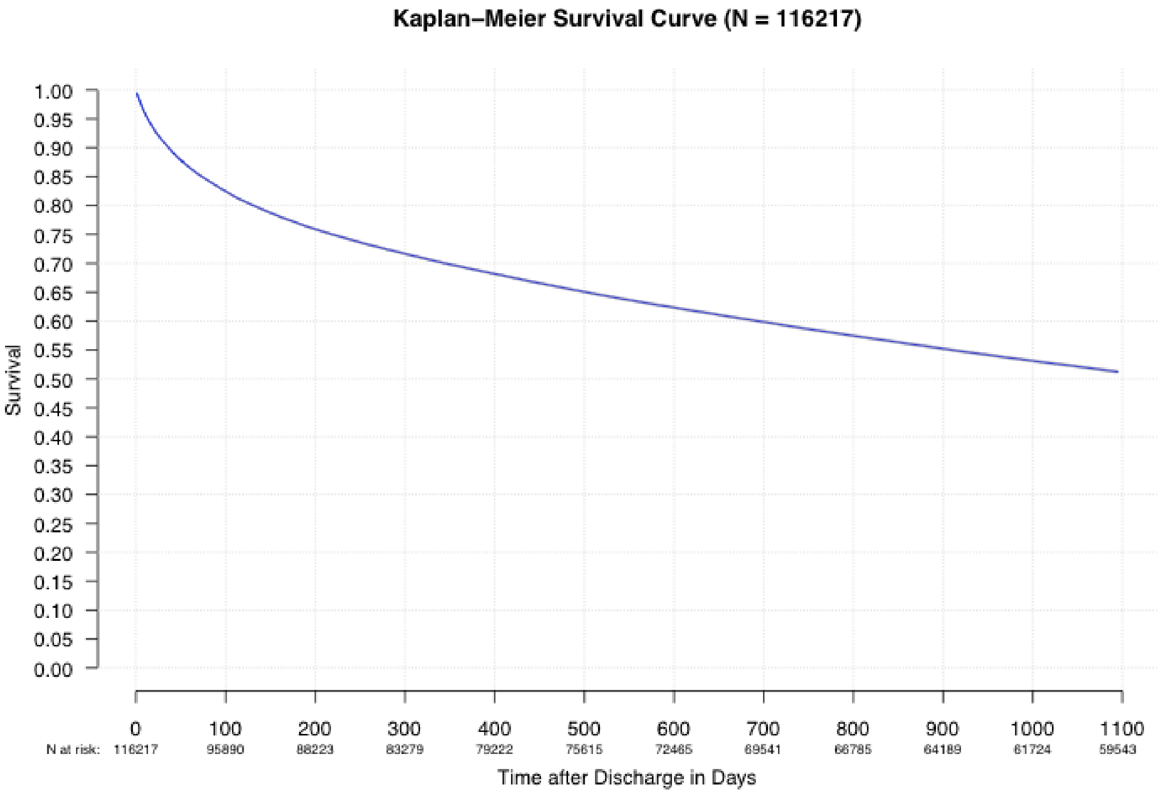

© 2021 Fleischmann-Struzek C et al. *JAMA Network Open.*

eFigure 3. Hazard Functions for Death for All Patients With Sepsis, Patients With Severe and Nonsevere Sepsis, Patients Treated in the ICU and Not Treated in the ICU, Patients with Sepsis According to Preexisting Impairments and by Age Groups

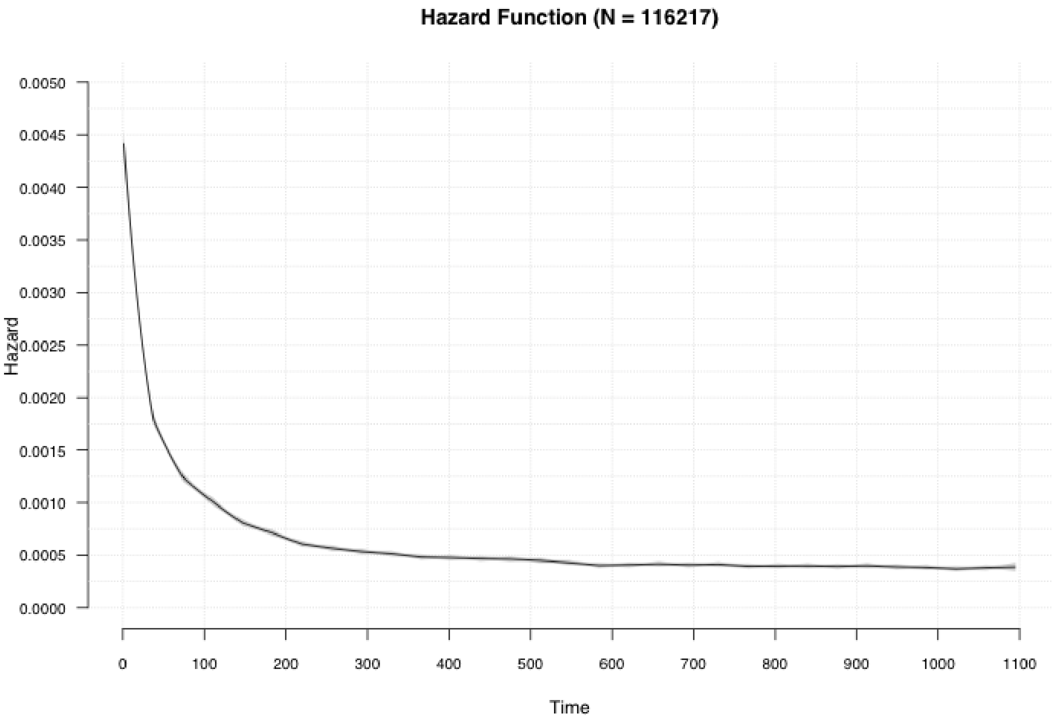

(a)

© 2021 Fleischmann-Struzek C et al. *JAMA Network Open.*

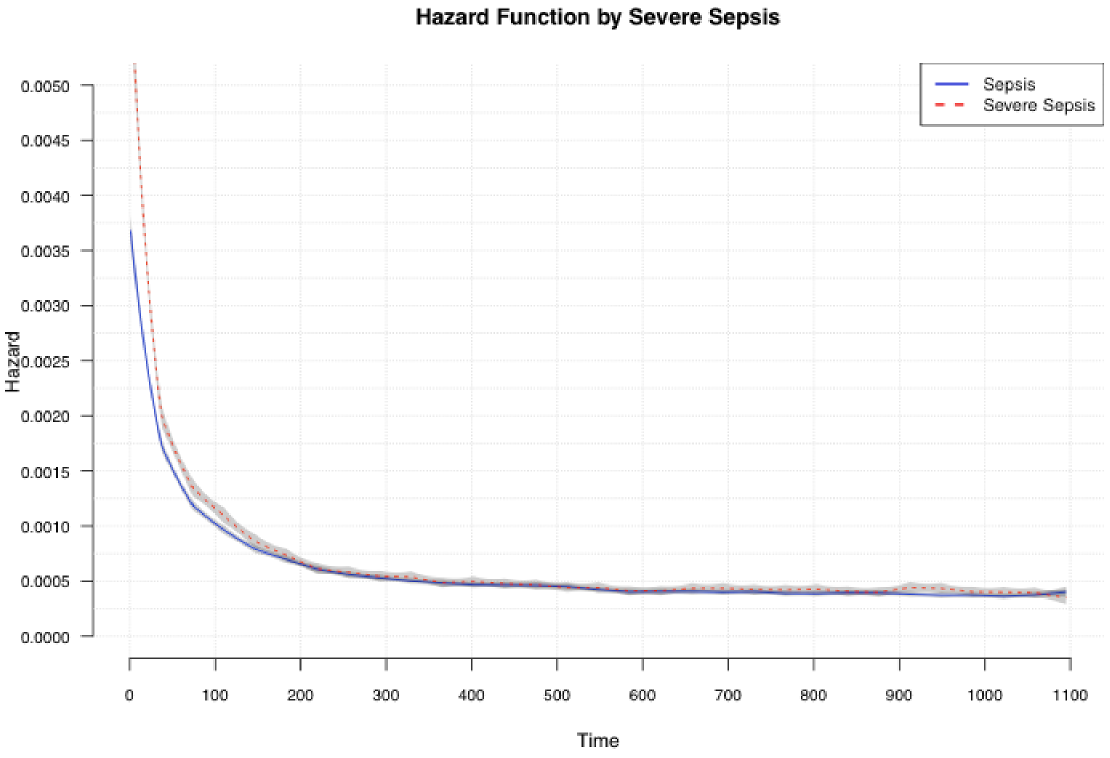

(b)

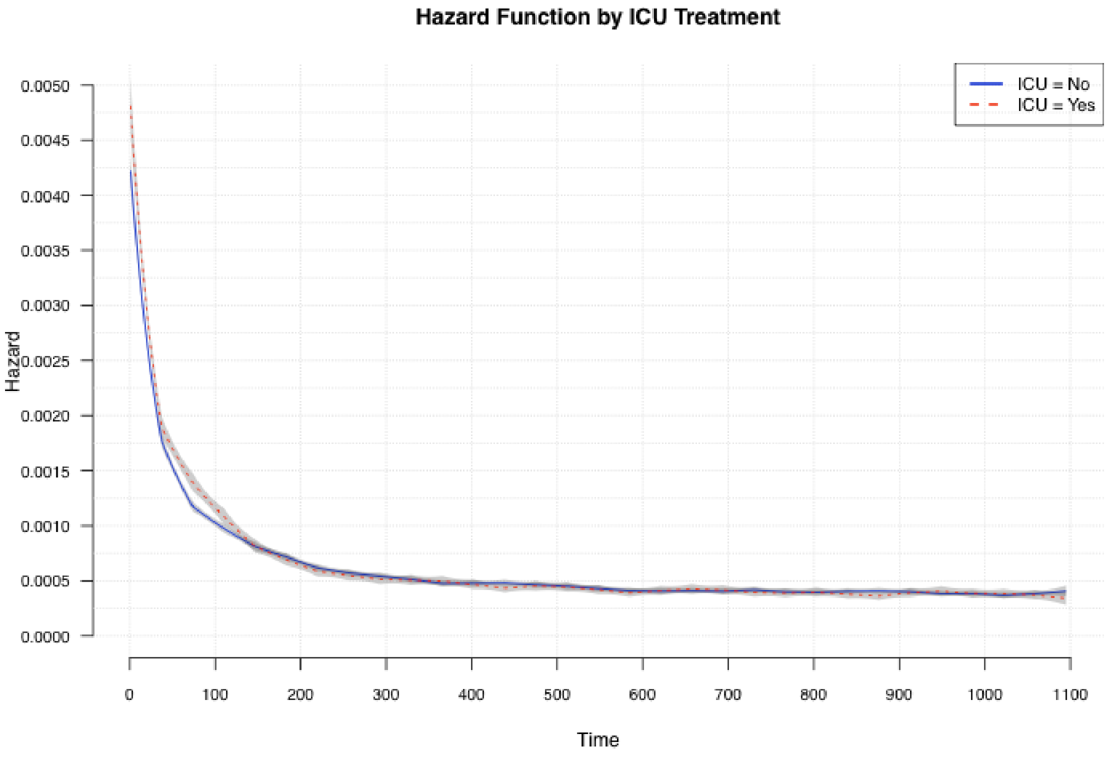

(c)

© 2021 Fleischmann-Struzek C et al. *JAMA Network Open.*

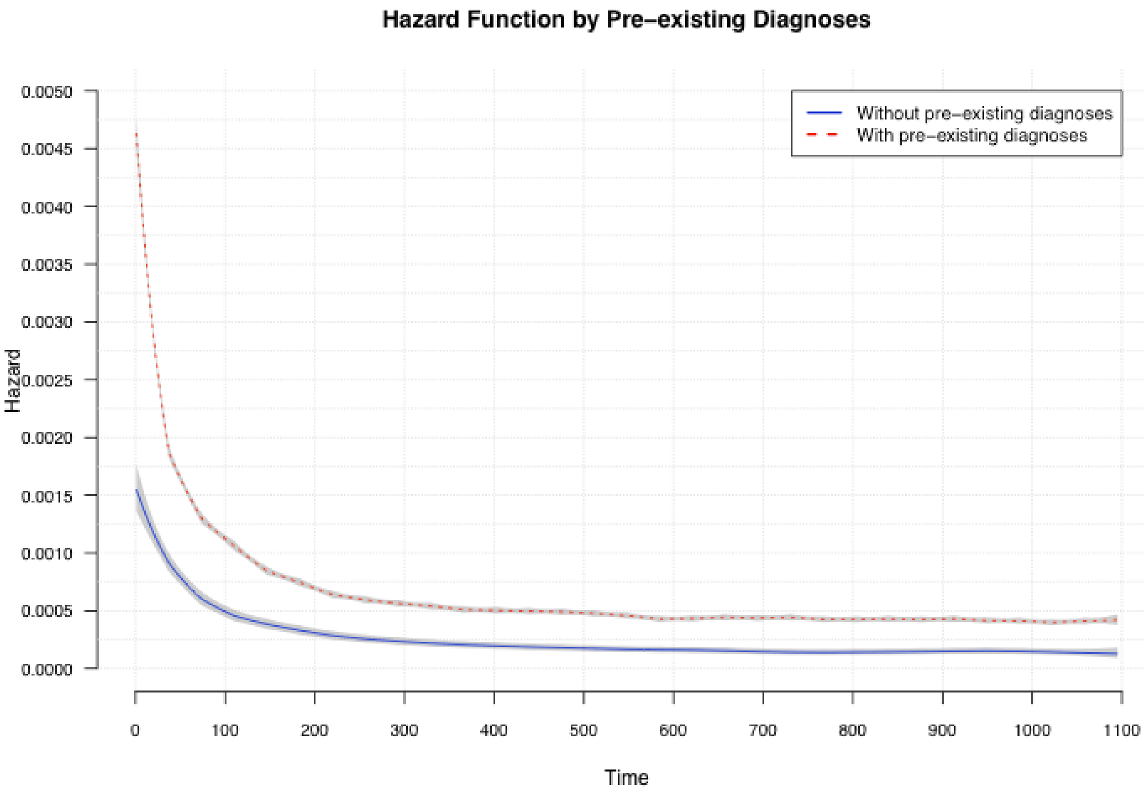

(d)

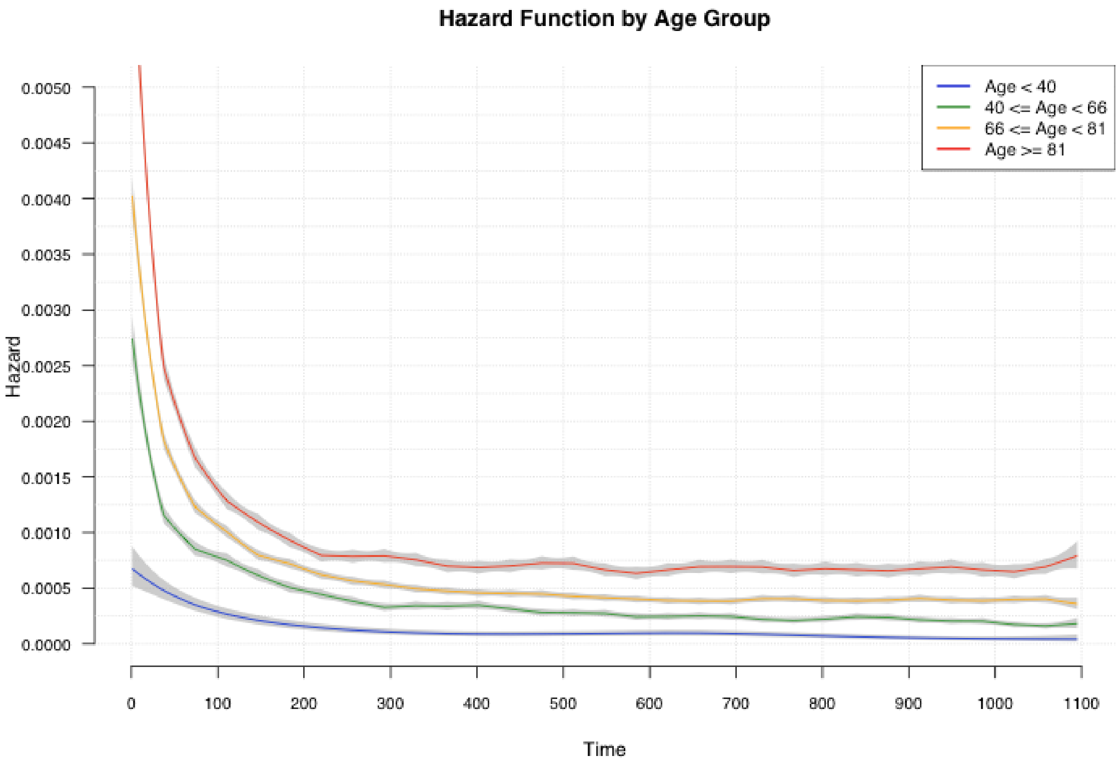

(e)

© 2021 Fleischmann-Struzek C et al. *JAMA Network Open.*
